# Maintaining social contacts: The physiological relevance of organelle interactions

**DOI:** 10.1016/j.bbamcr.2020.118800

**Published:** 2020-11

**Authors:** Beatriz S.C. Silva, Laura DiGiovanni, Rechal Kumar, Ruth E. Carmichael, Peter K. Kim, Michael Schrader

**Affiliations:** a College of Life and Environmental Sciences, Biosciences, University of Exeter, Exeter EX4 4QD, Devon, UK; b Program in Cell Biology, The Hospital for Sick Children, Toronto, ON, M5G 0A4, Canada; c Department of Biochemistry, University of Toronto, Toronto, ON, M5S 1A8, Canada

**Keywords:** ACBD, acyl-CoA binding domain containing protein, ABCD1, ATP binding cassette subfamily D member 1, ATF6α, activating transcription factor 6α, ATP, adenosine triphosphate, CRAC, calcium release-activated channel, DGAT2, diacylglycerol O-acyltransferase 2, DMT1, divalent metal transporter 1, DRP1, dynamin-related protein 1, ER, endoplasmic reticulum, ERMES, ER-mitochondria encounter structure, FFAT, two phenylalanines (FF) in an acidic tract, IMM, inner mitochondrial membrane, INF2, inverted formin 2, IP_3_R, 1,4,5-trisphosphate receptor, MAM, mitochondria-ER association membrane, MCS, membrane contact site, Mdm, mitochondrial division and maintenance, MFF, mitochondrial fission factor, MIGA2, Mitoguardin 2 protein, MIRO1, mitochondrial Rho GTPase 1, NPC1, Niemann-Pick C1 protein, Num1, nuclear migration protein 1, OMM, outer mitochondrial membrane, ORP, oxysterol-binding protein related protein, OSBP, oxysterol-binding protein, Osh, oxysterol-binding protein homology, PBD, peroxisome biogenesis disorder, PEX, peroxin (peroxisome biogenesis factor), PI(4)P, phosphatidylinositol-4-phosphate, PI(4,5)P_2_, phosphatidylinositol-4,5-bisphosphate, PLIN5, perilipin 5 protein, PM, plasma membrane, PPV, pre-peroxisomal vesicle, PTPIP51, protein tyrosine phosphatase interacting protein 51, ROS, reactive oxygen species, STARD3, StAR related lipid transfer domain containing protein 3, TAG, triacylglycerol, TBC1D15, TBC1 domain family member 15, TGN, trans-Golgi network, TMEM135, transmembrane protein 135, VAP, vesicle-associated membrane protein (VAMP)–associated protein, VLCFA, very long-chain fatty acid, VPS13, vacuole protein sorting-associated protein 13, Acyl-CoA binding domain containing protein, Peroxisomes, Mitochondria, Lipid metabolism, Membrane contact sites, FFAT motif

## Abstract

Membrane-bound organelles in eukaryotic cells form an interactive network to coordinate and facilitate cellular functions. The formation of close contacts, termed “membrane contact sites” (MCSs), represents an intriguing strategy for organelle interaction and coordinated interplay. Emerging research is rapidly revealing new details of MCSs. They represent ubiquitous and diverse structures, which are important for many aspects of cell physiology and homeostasis. Here, we provide a comprehensive overview of the physiological relevance of organelle contacts. We focus on mitochondria, peroxisomes, the Golgi complex and the plasma membrane, and discuss the most recent findings on their interactions with other subcellular organelles and their multiple functions, including membrane contacts with the ER, lipid droplets and the endosomal/lysosomal compartment.

## Highlights

• Membrane contact sites (MCSs) physically and functionally link organelles.
• MCSs are ubiquitous, diverse and dynamic.
• Mitochondria, ER, peroxisomes, Golgi and plasma membrane all form numerous MCSs.
• MCSs facilitate intracellular signalling, lipid and metabolite transfer.
• MCSs are important for organelle dynamics, division and inheritance.

## Graphical abstract

**Figure:**
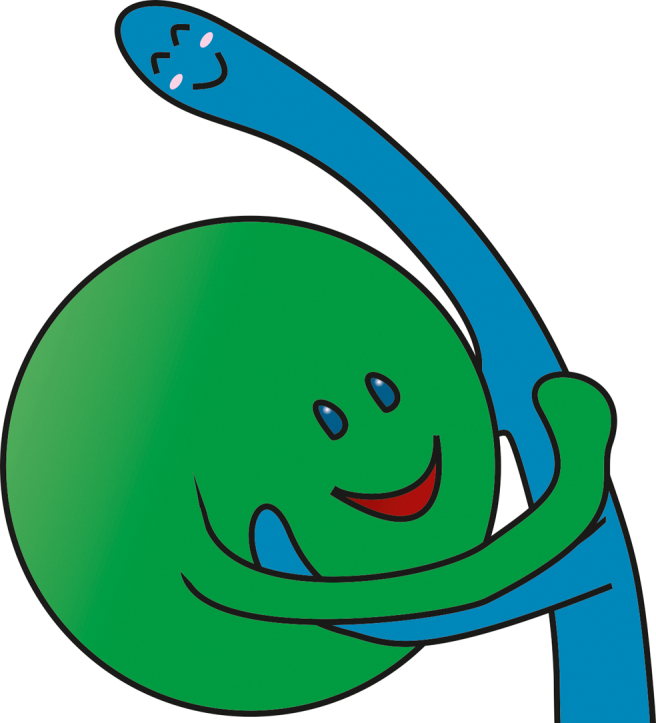

## 1 Introduction

Membrane-bound organelles in eukaryotic cells do not function as isolated entities. They form a “social network” within the cell and cooperate to coordinate and facilitate metabolic and other cellular functions. It is now evident that a coordinated interplay is often mediated by inter-organelle membrane contacts, which bring organelles in close apposition [1]. This review was written in times of the COVID-19 pandemic, where we more than ever value social contacts, and appreciate their importance for efficient communication and the maintenance of production chains. We also understand that “social distancing” and “self-isolation” are mechanisms for protection. In analogy, organelles can form so called membrane contact sites (MCSs), which facilitate the transfer of metabolites, lipids and proteins to fuel cooperative metabolic pathways, to efficiently exchange information for cellular signalling/communication, or to hold organelles in a specific location within the cell. Many membrane contacts require dynamic regulation, as they are not permanently required and need to be adapted to the changing needs of the cell. Thus, organelles can keep “social distance”, and can even “self-isolate”. An intriguing example is the assembly of actin filament cages at damaged mitochondria to prevent contact and fusion with neighbouring populations [2].

The research field of membrane contacts and organelle interaction is rapidly growing [[3], [4], [5]]. It is becoming clear that many, if not all, organelles form MCSs [6,7]. MCSs are formed by interacting proteins (or lipids) which function as tethers to bridge the opposing organelle membranes. The term ‘MCS’ generally describes a region of physical interaction between two organelles, which impacts on organelle function. However, ‘non-classical’ types of MCSs exist (e.g. between internal organelle membranes), and considerable variation in their composition, size, distance between organelles and stability have been described [3]. Guidelines have been delineated to define MCSs and their tethers [8,9], and new approaches are being developed to study and quantify MCSs, and to distinguish them from stochastic interactions [[10], [11], [12]]. Consequently, new contact sites are being discovered and the number of tethers and proteins associated with MCS is constantly expanding [9]. MCS resident proteins include molecular tethers, proteins involved in the transfer of small molecules (e.g. ions and lipids), as well as regulatory components. Many tether proteins appear to have additional functions (e.g. in lipid transfer) and localize to multiple MCSs (see Table 1). Current research is now focusing on the regulation of MCSs and their physiological functions, and it is becoming evident that MCSs are central to cell physiology and impact on human health and disease, thus changing our current understanding of disease pathology [[13], [14], [15], [16], [17], [18], [19]].

**Table 1:** Organelle interaction, MCS components and physiological role. ABCD1/3, ATP-binding cassette sub-family D member 1/3; ACBD2/4/5, acyl-coenzyme A binding domain containing protein 2/4/5; ACSL1, long chain fatty-acid-CoA ligase 1; AMPK, 5' AMP-activated protein kinase; ATF6α, activating transcription factor 6α; DGAT2, diacylglycerol O-acyltransferase 2; DMT1, divalent metal transporter 1; ENDO, endosome; ER, endoplasmic reticulum; ERMES, ER-mitochondria encounter structure; FAPP1, phosphatidyl-four-phosphate-adaptor-protein-1; FATP1, fatty acid transporter protein 1; GRP75, glucose regulated protein 75; INF2, inverted formin 2; IP3R, 1,4,5-trisphosphate receptor; LD, lipid droplet; LYS, lysosome; MAVS, mitochondrial anti-viral signalling protein; MIGA2, mitoguardin 2 protein; MITO, mitochondria; NLRP3, nucleotide-binding oligomerization domain-like receptor 3; OSBP1/ORP5/8/9/10/11, oxysterol-binding protein/OSBP-related protein family; PEX, peroxin; PI(4,5)P2, phosphatidylinositol-4,5-bisphosphate; PLIN5, perilipin 5 protein; PM, plasma membrane; PO, peroxisome; STARD3, StAR related lipid transfer domain containing protein 3; STIM1, stromal interaction molecule 1; TBC1D15, TBC1 domain family member 15; TMEM135, transmembrane protein 135; VAP, vesicle-associated membrane protein; VDAC, voltage dependent anion channel; VPS13, vacuole protein sorting-associated protein 13. MCS proteins are mammalian unless otherwise stated. * MCS components listed are those mentioned in the text; this is not a complete list of all MCS components identified so far.

Organelles (MCS)	MCS components*	Physiological role	References
Mitochondria – endoplasmic reticulum	SigmaR1 (MITO), SEL1L (ER)	Regulation of mitochondrial fission	[50]
	Spire1C (MITO) – INF2 (ER) tether		[[45], [46], [47]]
	IP_3_R (ER) – GRP75 – VDAC (MITO) tether	Transfer of Ca^2+^ between ER and MITO; Ca^2+^ signalling via MITO-ER contacts	[56,57]
	PDZD8 (MITO/LYS?) – Unknown protein (ER) tether	Dendritic Ca^2+^ homeostasis in mammalian neurons?	[60,62]
	Mmm1, Mdm12 and Mdm34 (ERMES, MITO), VPS13 *(yeast)*	Lipid transfer; phospholipid synthesis	[33,70,71]
	VPS13A (MITO) – VAP (ER)	Lipid transfer	[73,127]
	PTPIP51 (MITO) – ORP5/8 (ER)	Transport of phosphatidylserine from the ER to MITO	[78,79]
	NLRP3 (ER) – MAVS (MITO)	Immune signalling and inflammation	[[82], [83], [84], [85]]
	PTPIP51 (MITO) – VAPB (ER) tether	Autophagosome formation/autophagy	[[87], [88], [89]]
Mitochondria – lysosome	Tethers unknown	Regulation of mitochondrial dynamics/fission	[41]
	STARD3 (LYS) – tether?	Cholesterol transport to MITO (compensatory mechanism for impaired LYS-ER cholesterol transport)	[109]
	Regulated by RAB7, TBC1D15 (binds FIS1 at MITO)		[103,105,106]
	DMT1 (ENDO/LYS, MITO)	Iron transport from ENDO/LYS to MITO	[[119], [120], [121], [122]]
Mitochondria – lipid droplets	SNAP23, Unknown tether	Lipid transfer between LD and MITO for mitochondrial β-oxidation; energy metabolism	[124,126]
	Regulated by AMPK		[124]
	DGAT2 (ER/LD), FATP1 (ER)	LD expansion and biogenesis	[128,133,134]
	Unknown protein (MITO) – PLIN5 (LD)	Regulation of LD hydrolysis	[129,[135], [136], [137]]
	MIGA2 (MITO) – unknown protein (LD), VAPB (ER) (triple contact site?)	Lipogenesis	[139]
Peroxisomes– endoplasmic reticulum	ACBD4/5(PO) – VAPA/B (ER) tether, ACSL1	Coordination of fatty acid β-oxidation (PO) and elongation (ER); lipid transfer for ether-phospholipid synthesis; (phospho)lipid transfer for PO biogenesis (PO membrane expansion; regulation of PO positioning and mobility)	[[149], [150], [151]]
		Lipid synthesis for virus replication	[171]
	PI(4,5)P_2_ (PO) – *E*-SYTs	Transport of cholesterol	[207]
	ABCD3 (PO) – ATF6α (ER) tether	Regulation of ER stress; control of cellular stress response	[167]
	Pex3 (PO/ER) – Inp1 tether *(yeast)*	PO inheritance in yeast; control of PO abundance	[147,148]
	Pex24, Pex32 (ER) – Pex11 (PO) tether *(yeast)*	PO biogenesis and proliferation; positioning at the cell cortex; proper segregation to mother cells and buds	[146]
Peroxisomes – lipid droplets	ABCD1 (PO) – M1 spastin (LD) tether, ESCRTIII proteins IST1 and CHMP1B (LD)	Fatty acid trafficking between LDs and PO; lipolysis	[178]
Peroxisomes – mitochondria	Pex11 (PO) – Mdm34 (ERMES, MITO) *(yeast)*	Metabolic signalling?	[164]
	Pex34(PO), Fzo1 (MITO) tether *(yeast)*	Regulation of fatty acid β-oxidation in yeast (metabolite transfer)	[7]
	ACBD2 (PO/MITO)	Promotion of steroid biosynthesis (Leydig cells)	[193]
Peroxisomes – lysosomes/endosomes	PI(4,5)P_2_ (PO) – SYT7 (LYS) tether	Cholesterol transport from LYS to PO	[207]
	TMEM135 (PO)	Cholesterol transport; intracellular cholesterol distribution; regulation of ciliogenesis (cholesterol dependent)	[204]
	PxdA (ENDO) *(fungi)*	PO movement via endosome ‘hitch-hiking’	[213,214]
	Pex3 (PO - vacuole) tether *(yeast)*	PO growth/expansion; lipid transfer?	[146]
Golgi complex – endoplasmic reticulum	PI(4)P (TGN) – OSBP1/ORP9/10/11 – VAP (ER)	Direct transport of sterols	[20,219,[222], [223], [224]]
	PI(4)P (TGN) – CERT – VAP (ER)	Translocation of ceramide from the ER to TGN	[218,226]
	PI(4)P (TGN) – FAPP1 – SAC1 (ER) – VAP (ER)	PI4P homeostasis	[228]
Plasma membrane – endoplasmic reticulum	Osh2/3/6/7 (PM) Osh2/3 (PM) – VAP (ER) tether (?) *(yeast)*	Transport of sterols from the ER to the PM in yeast	[233]
	Scs2/Scs22 (ER), Sac1 (ER), Ist2 (ER), Tcb1/2/3 (ER) *(yeast)*	Phosphoinositide metabolism in yeast	[238]
	NIR2 C2CD2L/TMEM24	Phosphatidylinositol transport between the ER and the PM.	[239,240]
	ORAI1 (PM) – STIM1 (ER)	Maintenance of Ca^2+^ homeostasis; CRAC channel activation and Ca^2+^ entry	[243]
Plasma membrane – mitochondrian	PI(4,5)P_2_ (PM) – Num1 – Mdm36 (MITO)/cardiolipin (MITO)/Scs2 (ER) tether (=MITO-PM-ER triple contact) (yeast)	MITO inheritance; regulation of MITO distribution in yeast	[247,249]
	Mmr1 (MITO/ER) *(yeast)*		[254]
	Mfb1 (MITO) *(yeast)*		[255]
	MFN1 (MITO) – PKCζ (PM) tether (?)	Epithelial-mesenchymal transition (EMT) in mammals.	[256]
	Tethers unknown	Regulation of Ca^2+^ influx; mitochondrial Ca^2+^ import	[257]

The ER has long been the focus of MCS research [4,[20], [21], [22]], but MCS between other subcellular organelles have also been discovered, including mitochondria, peroxisomes, lipid droplets and lysosomes [[23], [24], [25], [26], [27]]. In this review, we will provide a comprehensive overview of the physiological relevance of organelle contacts. We will place mitochondria, peroxisomes, the Golgi complex and the plasma membrane in the centre of our review, and will summarize and discuss recent findings on their interaction with other subcellular organelles and their physiological role, including membrane contacts with the ER, lipid droplets and the endosomal/lysosomal compartment. We particularly focus on organelle interactions in mammalian/human cells, but where appropriate also refer to recent discoveries in yeast.

## 2 Mitochondrion-organelle interactions and their physiological relevance

### 2.1 Mitochondria-ER contacts

The endoplasmic reticulum (ER) is one of the most extensively studied and largest organelles in the cell. The ER is involved in many functions including protein and membrane lipid synthesis and transport, requiring it to communicate with other intracellular organelles including the Golgi apparatus, lysosomes, peroxisomes and mitochondria [4,28,29]. ER-mitochondria connections were first reported over 60 years ago in *Fundulus heteroclitus* by electron microscopy [30] and have been extensively characterised since. Electron tomography studies have shown that the ER-mitochondria distance in mammalian cells can be as close as ~10 nm at the smooth ER and ~25 nm at the rough ER [31], forming contacts at specialized domains called mitochondria-ER association membranes (MAMs), which are essential for cooperative functions like lipid transfer and calcium signalling [32].

**Fig. 1:**
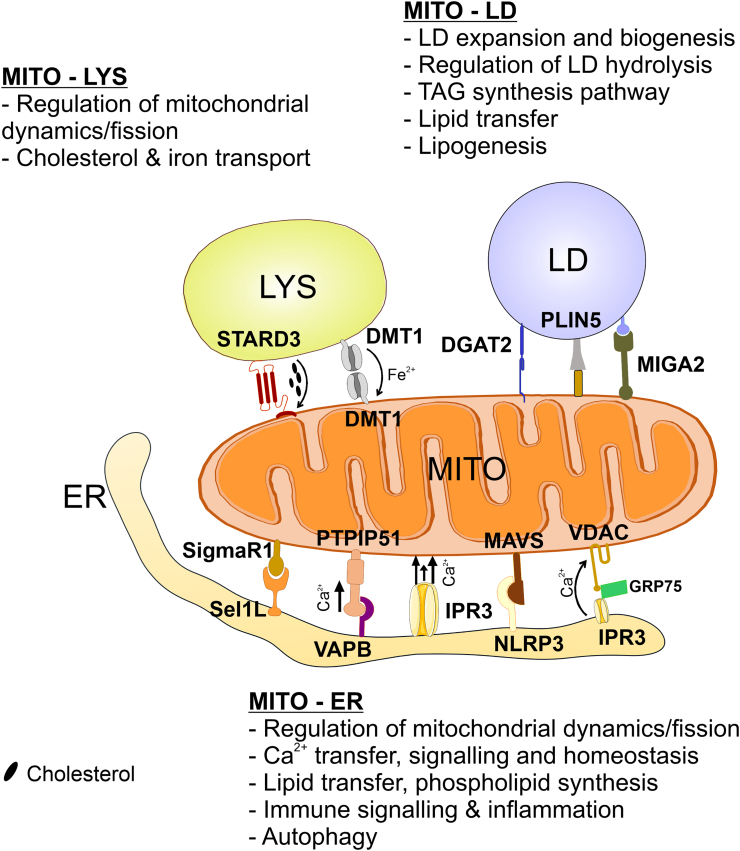
Schematic overview of mitochondrion-organelle interactions and their physiological relevance. DGAT2, Diacylglycerol O-acyltransferase 2; DMT1, Divalent Metal Transporter 1; ER, Endoplasmic Reticulum; GRP75, Glucose-Regulated Protein 75; IPR3, 1,4,5–triphosphate receptor; LD, Lipid Droplet; LYS, Lysosome; MAVS, Mitochondrial Antiviral-signalling protein; MIGA2, Mitoguardin 2 protein; MITO, Mitochondrion; NLRP3, Nucleotide-binding oligomerization domain-like receptor protein 3; PLIN5, Perilipin-5 protein; PTPIP51, Protein Tyrosine Phosphatase Interacting Protein 51; STARD3, Star-related lipid transfer protein 3; VAPB, Vesicle-Associated Membrane Protein (VAMP)-associated Protein B; VDAC, Voltage-Dependent Anion-selective Channel; VPS13A, Vacuole Protein Sorting-associated protein 13 isoform A.

ER-mitochondria tethering is best studied in the yeast system, where studies show that four proteins (Mmm1, Mdm10, Mdm12 and Mdm34) form a complex which connects the ER and mitochondria, which is often referred to as ERMES (ER-mitochondria encounter structures) [33,34]. ER-mitochondria contacts are reported to be important for multiple cellular functions, such as mitochondrial fission, Ca^2+^ signalling, lipid transport, energy metabolism, phospholipid synthesis, autophagy, immune signalling, glucose homeostasis, insulin signalling and inflammation [4,13] (Table 1; Fig. 1). ER-mitochondria contacts are crucial for normal physiological cell function, with miscommunication between the ER and mitochondria leading to diseases such as metabolic and neurodegenerative disorders [14,29,35]. For example, it was recently shown that increasing ER-mitochondria contacts with an artificial linker results in extended lifespan in a *Drosophila* model of Alzheimer's disease, suggesting modulating ER-mitochondria contact sites may be a new step to therapeutic strategies [36].

#### 2.1.1 Mitochondria-ER contacts regulate mitochondrial fission

Mitochondrial division is essential for cellular functions such as homeostasis of metabolism, mitochondrial quality control, and to regulate size, number, morphology and distribution of mitochondria in cells [37]. Impairment or dysfunction in mitochondrial fission has been directly linked to neurodegenerative and metabolic disorders [38,39]. Mitochondrial fission (as well as peroxisomal fission) (see Section 3.1.1) is controlled by large GTPases such as Dynamin related protein-1 (DRP1/DNML1). DRP1 is known to be recruited to mitochondria/peroxisomes by adaptor proteins such as MFF (Mitochondrial Fission Factor) [40]. ER-mitochondria contacts are important in the selection of mitochondrial fission sites [41], and mitochondrial fission and fusion events are spatially coordinated at ER-mitochondria MCSs [42]. Fission involves oligomerization of DRP1 at ER-mitochondria constriction sites, where DRP1 forms ring-like structures around the mitochondrion. ER tubules wrap around the mitochondria at constriction sites, where the GTPase activity of DRP1 results in conformational changes in the oligomer to cut the membrane at the constriction site which leads to the fission of mitochondria [41,43]. In addition, to ensure daughter mitochondria both inherit mitochondrial DNA (mtDNA), mtDNA nucleoids are recruited to ER-mitochondria contacts prior to constriction or DRP1 recruitment [44]. These contacts promote mtDNA synthesis in an ER tubule-dependent manner, resulting in spatial and temporal coordination of mitochondrial division and mtDNA replication for accurate segregation of nascent mtDNA.

Actin-mediated contractile forces at ER-mitochondria contact sites also promote fission, enhancing the recruitment of DRP1 and driving initial membrane constriction as a result of actin polymerization mediated by the ER-localised protein inverted formin 2 (INF2) and the mitochondria-anchored actin nucleator Spire1C [45,46] (Table 1). INF2 knockdown leads to elongated mitochondria, as a result of reduced fission, while its overexpression causes actin filament aggregation at ER-mitochondria contacts, which is the site of INF2 activation [47]. Mutations in INF2 lead to Charcot-Marie-Tooth disease [48], suggesting deregulation of mitochondrial fission at ER-mitochondria contacts could play a role in the pathophysiology of this disease. Interestingly, a recent study has proposed that PI(4)P-containing vesicles derived from the trans-Golgi network are also recruited to ER-mitochondria fission sites to facilitate the final scission of the mitochondrial membrane. This, in addition to the presence of lysosomes at mitochondria sites of fission (see Section 2.2.1) raises the possibility that mitochondrial fission may be regulated by three- or even four-way contact sites [49].

A recent study has implicated components of the ER-associated degradation (ERAD) quality control pathway in regulating mitochondrial dynamics via ER-mitochondria contacts [50]. In brown adipocytes, loss of the ER-resident ERAD protein Sel1L prevents the mitochondrial fission usually stimulated by cold stress, resulting in enlarged ‘megamitochondria’ with impaired metabolic functions. Counterintuitively for a fission defect, loss of Sel1L actually increased ER-mitochondrial contacts, via reduced degradation of the MAM protein SigmaR1, leading to long ER tubules that even appeared to perforate mitochondria, and may represent stalled fission or accelerated fusion events. Therefore, depending on the context and proteins involved, ER-mitochondria contacts can either positively or negatively induce mitochondrial fission (Table 1).

#### 2.1.2 Mitochondria-ER contacts regulate Ca^2+^ signalling

Calcium ions are one of the most ubiquitous secondary messengers in the cell and are involved in a complex and dynamic variety of physiological functions, which includes signal transduction, muscle contraction, secretion of proteins, secretion of hormones, gene expression, induction of various forms of cell death (necrosis, apoptosis and autophagy) and neuronal function [51]. Ca^2+^ uptake and release are crucial functions of both mitochondria and the ER, meaning they must cooperate to facilitate signalling and maintain homeostasis. The inner mitochondrial membrane (IMM) has selective gated Ca^2+^ ion channels, which allows the Ca^2+^ ions to enter the mitochondrial matrix via the mitochondrial calcium uniporter (MCU, previously CCDC109A) [52]. The ER is the major site of Ca^2+^ storage in the eukaryotic cell, with internal concentrations of approximately 1 mM, which is relatively close to the extracellular concentration, although significant heterogeneity in Ca^2+^ levels exists between different regions of the ER [51,53].

In the resting state, cytosolic Ca^2+^ is maintained at approximately 100 nM, depending on cell type. A wide variety of stimuli induce transient Ca^2+^ influx into the cytosol to propagate signalling, which requires subsequent uptake and storage of excess Ca^2+^ to return to the homeostatic levels [32]. Mitochondrial accumulation of Ca^2+^ following release of Ca^2+^ from the intracellular reservoir of the ER, as well as local Ca^2+^ signalling between the two organelles, requires close ER-mitochondria apposition [54] and involves the Ca^2+^ release channel IP_3_R (1,4,5-trisphosphate receptor), a transmembrane protein located at the ER and Golgi membranes [55]. A recent study has shown IP_3_R has a role in maintaining ER-mitochondria contacts, independent of its Ca^2+^ transfer function. While the different isoforms of IP_3_R can all support the formation of ER-mitochondria contacts, IP_3_R isoform 2 is the most efficient at delivering Ca^2+^ to mitochondria from the ER [56] (Table 1; Fig. 1).

Mechanistically, cooperative Ca^2+^ transfer between the ER and mitochondria is facilitated by the cytosolic chaperone glucose-regulated-protein 75 (GRP75) which forms a tethering complex by simultaneously binding IP_3_R and the mitochondrial porin voltage-dependent anion channel (VDAC) in the outer mitochondrial membrane (OMM), with knockdown of GRP75 destroying the functional Ca^2+^ coupling between the ER and mitochondria [57] (Table 1). Physiologically, GRP75–mediated ER-mitochondria tethering has recently been shown to promote regeneration of damaged axons in neurons by increasing mitochondrial Ca^2+^ which in turn increases ATP production [58,59]. In mammalian neurons, PDZD8 has been proposed as an alternative ER-mitochondria tether that is required for the mitochondrial uptake of Ca^2+^ following its stimulated release from the ER in response to synaptic activation, to regulate cytosolic Ca^2+^ dynamics [60] (Table 1). However, despite initially being characterised as a functional orthologue of the yeast ERMES component Mmm1, there is increasing evidence that PDZD8 may be only distantly related to Mmm1 [61], and may in fact predominantly localize to ER-late endosome/lysosome contacts [62].

The role of ER-mitochondria MCSs in activity-dependent, responsive Ca^2+^ transfer raises the intriguing question of how these MCSs are regulated by external stimuli. A recent high-throughput drug screen using split luciferase complementation as a quantitative readout of ER-mitochondria contacts identified a number of G-protein coupled receptor (particularly β-adrenergic receptor) agonists that increased the extent of ER-mitochondria contacts and thus mitochondrial Ca^2+^ uptake [63]. While this increase in contacts seemed to depend on a rise in cytosolic Ca^2+^ and an increase in actin polymerization induced by receptor activation, how this mechanistically couples to ER-mitochondria contacts to regulate their formation remains to be seen.

#### 2.1.3 Mitochondria-ER contacts facilitate lipid transport

The ER is known as the “lipid hub” of the cell and participates in the transport of the majority of the lipids to other organelles, such as mitochondria, which cannot synthesize all of the lipids they need to function and thus depend on transfer of lipids and precursors from the ER [64,65]. Tethering of the ER and mitochondria is crucial for cell growth because it facilitates the transport of membrane lipids to mitochondria, which is essential for mitochondrial function as well as expansion of the mitochondrial network [66] (Fig. 1). In both yeast and mammals, the major lipids which are transported from ER to mitochondria are the phospholipids phosphatidylserine (PS), phosphatidylcholine (PC), phosphatidylethanolamine (PE) and phosphatidylinositol (PI), as well as sphingolipids and sterols [67]. In yeast, phosphatidic acid is also transferred from the ER to mitochondria where it is used to synthesize cardiolipin, although if/how this transfer occurs in mammals is unclear [68].

Lipid metabolism occurs in numerous organelles, and the differential localisation of enzymes involved in the same biosynthetic pathway necessitates close contacts for the bidirectional transfer of intermediates between different compartments including the ER and mitochondria (see also **3**.**3**.**1**). The experimental approaches to unravel the mechanism and molecules involved in the lipid transport between the ER and mitochondria for lipid metabolism have been extensively discussed [69]. The process of coordinated lipid synthesis between the ER and mitochondria varies between yeast and mammalian systems based on differences in synthesis pathways and enzyme localisations. Briefly, PS is produced in the ER – in mammals, this occurs from the precursors PC or PE in a reaction catalysed by PS synthase 1 (PSS1) and PS synthase 2 (PSS2) respectively, whereas in yeast, PS synthesis is catalysed by Pss1 from CDP-Diacylglycerol as a precursor. In both systems, ER-synthesised PS can then be transported to the mitochondria where it can be converted to PE by decarboxylation. Yeast also possess a Golgi-localised PS decarboxylase (Psdp2), meaning PE synthesis can occur in the Golgi as well as in mitochondria, in contrast to mammals [68]. Newly-synthesised PE can be transferred back to the ER from the mitochondria and/or Golgi, where it can be converted to PC by methylation or distributed to the cellular membranes – notably, the inter-organelle PS-PE shuttle requires ATP in mammalian cells whereas it can proceed independently of ATP in yeast, suggesting different mechanisms of lipid transfer between the opposing membranes in the two systems [67,68,70].

Lipid transport between organelles is well studied in the yeast system, where ERMES tethers are known to exchange the essential phospholipids [33], with the ERMES components Mmm1, Mdm12 and Mdm34 containing synaptotagmin-like mitochondrial lipid binding domains for non-vesicular lipid transport [71]. In yeast, the vacuole protein sorting-associated protein 13 (Vps13) localizes to numerous membrane contact sites, and can compensate for loss of ERMES subunits [70,72] (Table 1). VPS13 is conserved in mammalian cells and has two orthologues, VPS13A and VPS13C, which bind to ER-resident proteins via FFAT motifs and localize variously to ER-mitochondria (A), ER-lipid droplet (A & C) and ER-endosome (C) contact sites [73]. VPS13A contains a mitochondria-binding domain at the C-terminus, while its hydrophobic N-terminal domain can bind lipids and transport them between artificial membranes in vitro [73]. Loss of VPS13A results in neurodegeneration and misshaped erythrocytes, highlighting the importance of ER-mitochondria lipid transfer in physiological cell function (Table 1; Fig. 1). Additionally, the N-terminal domain of the autophagy protein ATG2, which can transfer lipids between the ER and autophagosome membrane [74,75], is homologous to that of VPS13A [73], raising the possibility that ATG2 might also be involved in lipid transport at ER-mitochondria and/or ER-lipid droplet MCSs.

While lipid transport between the ER and mitochondria is less well defined in the mammalian system, evidence suggests the potential involvement of oxysterol-binding protein (OSBP)-related proteins ORP5 and ORP8 in PS shuttling in mammals. While ORP5 and ORP8 are known to facilitate transfer of PS from the ER to the PM in counter exchange with PI(4)P at ER-PM contact sites [76,77], ORP5/ORP8 have also been shown to localize to mitochondria-ER contacts where their depletion resulted in mitochondrial morphology and respiration defects [78]. Given the role of ORP5/ORP8 in PS exchange at ER-PM contact sites, and localization to mitochondria-ER contact sites, an intriguing possibility is that ORP5/ORP8 also function to transport PS at mitochondria-ER MCSs [79].

#### 2.1.4 Mitochondria-ER contacts in immune signalling and inflammation

Inflammation is the biological response in body tissues which is induced by harmful external stimuli such as pathogens or irritants. ER-mitochondria contact sites have a major role in a number of immune regulatory processes, such as leukocyte migration, lymphocyte activation, sensitization to cell death, B and T cell homeostasis, and modulation of the cytotoxic anti-cancer response, which are all affected by impairment of ER and mitochondrial functions (for a detailed review, see [80]).

Inflammation in the body can be triggered by the activation of nucleotide-binding oligomerization domain-like receptors (NLRs), including NLRP3, which redistributes from the ER to the perinuclear region at ER-mitochondria contact sites upon its activation [80]. This relocation of NLRP3, via docking to the mitochondrial anti-viral signalling protein (MAVS) in response to cellular stress (such as reactive oxygen species (ROS) production) or viral infection, promotes cytokine release, suggesting ER-mitochondria contact sites play a role in the initiation of inflammation [81] (Table 1; Fig. 1). For molecular details of ER-mitochondria contacts involved in the innate and adaptive immune system, see [[82], [83], [84], [85]].

#### 2.1.5 Mitochondria-ER contacts in autophagy

Autophagy is the catabolic process that intrinsically degrades damaged cells and cytoplasmic proteins, prolonging their survival during nutrient starvation by engulfing, degrading and recycling intracellular components within specialized double-membrane vesicles known as autophagosomes. In recent years it has been shown that autophagosomes can form at ER-mitochondria contact sites in mammals, and these MCSs are required for this type of autophagosome formation [86]. The integral ER protein vesicle-associated membrane protein-associated protein B (VAPB) binds to the OMM protein, protein tyrosine phosphatase interacting protein 51 (PTPIP51), forming one of the tethering complexes linking the ER and mitochondria [87] (Table 1; Fig. 1). Dysregulation of the VAPB-PTPIP51 ER-mitochondria tether induces autophagy [88]. Overexpression of VAPB or PTPIP51 increased ER-mitochondria contacts and impaired autophagosome formation – this is specifically due to the tethering function of the proteins, as expression of an artificial tether was sufficient to reduce autophagosome formation, and required the ER-mitochondria Ca^2+^ transport function of the VAPB-PTPIP51 tether [88]. Autophagy is also reduced by the decreased transport of lipids from the ER to mitochondria, and subsequently the autophagosome, following the disruption of ER-mitochondria contacts [89].

Recent studies have also highlighted the involvement of another MAM-localised pathway in autophagosome assembly, by showing that during starvation the cytosolic SNARE protein Syntaxin 17 (STX17) translocates to MAMs where it recruits the pre-autophagosome proteins ATG14 and ATG5 [86]. Later, ATG14 interacts with PI3KR4/VPS15 kinase and the ER protein Beclin1 (BECN1), which also relocate to MAMs upon starvation, inducing the lipid kinase activity of the PI3KC3 complex, the first step of phagophore formation [90]. Under resting conditions, the OMM anti-apoptotic protein Bcl-2 suppresses autophagy by interacting with Activating molecule in BECN1-regulated autophagy protein 1 (AMBRA1) at the mitochondrial surface. In response to starvation, AMBRA1 dissociates from Bcl-2, freeing it to bind to BECN1 at ER-mitochondria contact sites to induce autophagy [91]. In tumour cells, the interplay between apoptosis and autophagy induction can also be regulated by the presence of the tumour suppressor p53 and the promyelocytic leukemia (PML) protein at MAMs. The interaction between p53 and PML in these ER-mitochondria appositions regulates the transfer of Ca^2+^ from the ER to the mitochondria, promoting Ca^2+^-dependent apoptosis [92,93]. The disruption of ER-mitochondria contacts also leads to an increase in mTOR-independent AMPK-dependent autophagic flux, which in turn leads to ER-mitochondria Ca^2+^ transfer inhibition, with AMPK present at the MAMs activating localised autophagy via BECN1 [94].

### 2.2 Mitochondria-lysosome contacts

Mitochondria and lysosomes are intricately interrelated organelles, best highlighted by the common dysfunction of both organelles seen in disease [[95], [96], [97], [98], [99]]. The most obvious connection between lysosomes and mitochondria is the role of lysosomes in the degradation of mitochondria through autophagy. This involves the engulfment of a mitochondrion by an autophagosome, followed by fusion of the autophagosome with lysosomes to acidify and degrade mitochondrial components. Similarly, mitochondria-derived vesicles have been shown to fuse directly with lysosomes [100]. However, beyond the degradative connection of lysosomes and mitochondria, multiple lines of evidence suggest mitochondria-lysosome interplay is involved in the normal functioning of both organelles. For example, impairment of mitochondrial function through deletion of mitochondrial proteins, or chemical inhibition of the electron transport chain, causes impaired lysosomal function [97]. In addition, the lysosomal biogenesis factor TFEB promotes mitochondrial biogenesis, as well as increasing the expression of oxidative phosphorylation enzymes [101], while inhibition of lysosomal acidification results in diminished basal and maximal mitochondrial oxygen consumption rates [102]. This evidence indicates that the normal functions of lysosomes and mitochondria are tightly linked, and thus are likely highly coordinated.

One possible mechanism of coordinating mitochondria and lysosome functions is through direct contact. MCSs between lysosomes and mitochondria have been observed using multiple methodologies [[6], [103]]. Importantly, many instances of lysosome-mitochondria contacts are seen to be independent of autophagy machinery, and temporal experiments visually tracing mitochondria and lysosomes have shown that lysosome-mitochondria contacts resolve without degradation of mitochondria [103]. This indicates that lysosome-mitochondria contacts can be involved in the non-degradative functions of lysosomes and mitochondria.

Lysosome-mitochondria contact has been shown to be regulated by Rab7, the master regulator of late endosome/lysosome dynamics. Rab7 is a GTPase whose localization to the late endosome/lysosome membrane is dependent on its nucleotide bound state. GDP-bound Rab7 (Rab7-GDP) is inactive and cytosolic, while GTP-bound Rab7 (Rab7-GTP) is active and recruited to the late endosome/lysosome membrane [103]. Active membrane-bound Rab7-GTP acts by binding and recruiting Rab effector proteins to the lysosome, which then function in facilitating lysosomal transport, fusion and organelle contact. Lysosomal dynamics are thus controlled through modulating the GTP bound state of Rab7, through guanine nucleotide exchange factors (GEFs) and GTPase activating proteins (GAPs).

Rab7-GTP can promote the formation of lysosome-mitochondria contacts. Overexpression of a non-hydrolysable and constitutively active Rab7 mutant, Rab(Q67L)-GTP, increased both the number and duration of lysosome-mitochondria contacts when compared to overexpression of wild type (WT) Rab7 [103]. In turn, lysosome-mitochondria contact termination is regulated by the GTPase activating protein (GAP) TBC1D15, which is recruited to mitochondria by the mitochondrial membrane protein FIS1 [[104], [105], [106]] (Table 1). The duration of lysosome-mitochondria contact events in TBC1D15−/− cells are significantly lengthened. Similarly, mutants of TBC1D15 which lack GAP activity (TBC1D15(D397A) and TBC1D15(R400K)) also have significantly extended lysosome-mitochondria contact duration. Importantly, the role of TBC1D15 on lysosome-mitochondria contact termination is dependent on TBC1D15 mitochondrial localization, as both knockout of FIS1 and overexpression of a FIS1 mutant unable to recruit TBC1D15 to mitochondria (FIS1(LA)) result in an increase in the number and duration of lysosome-mitochondria contacts [103]. This indicates that TBC1D15 acts on Rab7-GTP at sites of mitochondria-lysosome contact.

Together, these data suggest a model of lysosome-mitochondria contact regulation whereby Rab7-GTP promotes contact formation between lysosomes and mitochondria, while mitochondrial TBC1D15 hydrolyzes Rab7-GTP to terminate lysosome-mitochondria contact. What remains to be answered in this model is the identification of the proteins that physically tether lysosomes and mitochondria. Presumably, Rab7-GTP acts to promote lysosome-mitochondria contact through recruitment of an as-yet-unidentified effector protein which acts as a tether to mitochondria. Additionally, regulatory factors upstream of Rab7-GTP and TBC1D15 remain to be determined. What physiological conditions and protein machinery promotes the formation of Rab7-GTP to initiate lysosome-mitochondria contact? What promotes the mitochondrial recruitment of TBC1D15 to terminate lysosome-mitochondria contact? And, above all, what function does lysosome-mitochondria contact serve in coordinating the normal, non-degradative function of lysosomes and mitochondria?

#### 2.2.1 Mitochondria-lysosome contacts regulate mitochondrial dynamics

Mitochondria are extremely dynamic organelles that continuously undergo fission and fusion events to reorganize the mitochondrial network. How specific sites of mitochondria are designated for fusion or fission events is not clear, but mitochondrial MCSs with various organelles have been implicated in specifying fission sites. While many studies have focused on the contribution of the ER (see Section 2.1.1), lysosome contacts with mitochondria have also recently been shown to promote mitochondria fission [41] (Table 1). Lysosomes have been observed to localize to a large proportion of mitochondrial fission events (significantly higher than would be expected through random occurrence) [103,107], and are more prevalent than other organelles at mitochondrial fission events [103]. Furthermore, lysosomes were shown to be functionally involved at mitochondrial fission events as expression of non-hydrolysable Rab7(Q67L)-GTP resulted in decreased mitochondrial fission events, despite increased mitochondria-lysosome contact [103]. This suggests that lysosome-mitochondria contact termination is involved in promoting mitochondria fission events. However, the specific mechanism by which lysosomes contribute to mitochondrial fission is not known.

Similarly, lysosome-mitochondria contact sites have been implicated in regulating a third type of mitochondrial dynamics, mitochondria-mitochondria contact (inter-mitochondrial contact). Inter-mitochondrial contact is characterised by inter-mitochondria tethering and untethering without membrane fusion, ingeniously assessed using differential excitation of photoactivatable matrix probes in adjacent mitochondria. As with mitochondrial fission events, lysosomes were observed at a large proportion of inter-mitochondrial untethering events. Lysosome recruitment to inter-mitochondria contact sites was temporally coupled to untethering, whereby lysosome contact directly preceded mitochondria-mitochondria contact termination. Impairment of Rab7-GTP hydrolysis through expression of Rab7(Q67L)-GTP, TBC1D15(D397A) and FIS1(LA) extended the duration of inter-mitochondrial contact, implicating Rab7-GTP hydrolysis in inter-mitochondrial contact termination [107]. Interestingly, the ER was also seen at a large proportion of inter-mitochondria untethering events (as well as fission and fusion events), indicating that mitochondria, the ER and lysosomes may act in a triple-MCS to regulate inter-mitochondria untethering events. While the functional significance of inter-mitochondria contact has not been elucidated, inter-mitochondria contact was shown to increase in response to mitochondrial dysfunction (rotenone) and increased mitochondrial respiration (nutrient starvation). These stimuli may offer clues into the potential function of inter-mitochondria contact, and the role lysosomes may play at these sites of contact.

#### 2.2.2 Mitochondria-lysosome contacts regulate cholesterol transport

The endocytic pathway is involved in the transport of cholesterol from endocytosed low-density-lipoprotein (LDL) to the ER. Direct transport of cholesterol between lysosomes and the ER occurs at lysosome-ER contact sites and requires the transmembrane endosomal protein NPC1, which has been shown to interact with ER resident proteins ORP5 and Gramd1b [108,109]. Cells deficient in NPC1 display endosomal accumulation of cholesterol [110,111]. Interestingly, impairment of lysosome-ER cholesterol transport in NPC1-deficient cells also resulted in cholesterol accumulation in mitochondria, implicating the involvement of mitochondria in cholesterol trafficking from lysosomes [109,112].

Indeed, cholesterol transport from lysosomes to mitochondria has recently been shown to occur at lysosome-mitochondria contact sites as a compensatory mechanism for impaired lysosome-ER cholesterol transport [109]. Abolishment of lysosome-ER contacts through knockdown, knockout or chemical inhibition of NPC1 resulted in a reciprocal increase in lysosome-mitochondria contacts. Lysosome-mitochondria contacts were shown to be dependent on the endosomal sterol binding protein STARD3 which relocates from ER-lysosome to mitochondria-lysosome contact sites in NPC1-deficient cells [109] (Table 1; Fig. 1). Depletion of STARD3 in NPC1 mutant cells prevented the mitochondrial accumulation of cholesterol, indicating that STARD3-mediated lysosome-mitochondria contacts facilitate the transport of cholesterol to mitochondria [112]. While lysosome-mitochondria contacts were shown to dramatically increase upon lysosome-ER contact inhibition, knockdown of STARD3 reduced lysosome-mitochondria contacts to levels below that of WT cells, where lysosome-ER contacts were intact [109]. This suggests that lysosome-mitochondria contacts may function in cholesterol trafficking even at steady states, and are upregulated when there is an accumulation of cholesterol in lysosomes as a compensatory mechanism.

While STARD3 appears to be required for lysosome-mitochondria tethering and cholesterol transport, how STARD3 anchors to the mitochondria is not known. Also unclear is if and how Rab7 may regulate STARD3-mediated lysosome-mitochondria contact. Interestingly, cholesterol accumulation has been shown to increase the proportion of membrane-associated Rab7 [113]. Furthermore, overexpression of Rab7 or stabilization of Rab7-GTP have been shown to reduce global cholesterol accumulation in NPC1 mutant cells [114,115]. While the effect of promoting Rab7-GTP in NPC1 mutant cells was linked to rescuing lysosome-ER contact, increased Rab7-GTP during high cholesterol conditions may also act to rescue lysosomal cholesterol efflux through lysosome-mitochondria contact [115].

#### 2.2.3 Mitochondria-lysosome contacts mediate iron transport

Iron is essential for many cellular pathways, especially in mitochondria, where it is required for the biosynthesis of haem and iron-sulphur clusters [116]. However, iron is also extremely toxic to cells by producing hydroxyl radicals through the reaction of reduced Fe^2+^ with oxygen (Fenton reaction). Given the oxygen-rich cytosolic environment, the trafficking and storage of iron within the cell must be tightly controlled. The mechanism of iron uptake into cells through transferrin-mediated endocytosis is well established and terminates with the release of iron from the transferrin receptor through acidification of the endocytic vesicle. Iron within the endolysosomal system is then transferred to mitochondria, the major iron storing compartment in the cell, where it is assembled into iron-sulphur clusters. This transfer of endocytosed iron from the endolysosomal system to mitochondria has been suggested to be mediated by endosome/lysosome-mitochondria contact.

Transferrin-positive endosomes have been shown to contact mitochondria using confocal microscopy, electron microscopy and STORM imaging [117,118]. Temporal studies of this interaction have demonstrated contact occurs in brief “kiss and run” interactions. Contact between transferrin-positive endosomes and mitochondria was shown to mediate iron import into the mitochondria, as a mitochondrially-localised fluorescent metallosensor was immediately quenched following contact with transferrin-positive endosomes [117,118]. Divalent metal transporter 1 (DMT1) is a metal ion-proton cotransporter implicated in mediating iron trafficking between the endolysosomal compartment and mitochondria [119]. DMT1 localizes to the late endosome/lysosome membrane and is responsible for facilitating iron efflux from the endolysosomal compartment following endosome acidification [120]. Interestingly, DMT1 also localizes to the mitochondrial membrane and has been suggested to mediate mitochondrial iron uptake [121,122]. Studies have shown that iron uptake into mitochondria is decreased by DMT1 inhibition, while DMT1 overexpression enhances mitochondrial iron uptake [122]. However, while these results clearly indicate to the involvement of DMT1 in iron exchange between the endolysosomal system and mitochondria, it is difficult to differentiate the role of endolysosomal and mitochondrial DMT1 as methods used to assess involvement of DMT1 affect both populations.

A model of endosome/lysosome-mitochondria iron exchange has been proposed whereby endolysosomal DMT1, tasked with iron efflux, and mitochondrial DMT1, tasked with iron influx, associate at sites of endosome/lysosome-mitochondria contact to facilitate iron transport from endosomes to mitochondria (Table 1; Fig. 1). However, since DMT1 acts as a metal-H^+^ cotransporter [119], and the mitochondrial inter membrane space is an acidic environment, it would be expected that DMT1 would be involved in iron efflux from mitochondria, rather than influx. One hypothesis put forward to reconcile this with the proposed function of mitochondrial DMT1 in iron import is that protons effluxed with iron by endolysosomal DMT1 may create local domains of high acidity at the endosome/lysosome-mitochondria interface creating a local inward proton gradient at the mitochondria membrane to allow mitochondrial iron influx. This is supported by evidence showing an increase in mitochondrial iron import following acute incubation of mitochondria in an acidic environment [122]. Alternatively, mitochondrial DMT1 may function only in iron efflux from mitochondria and an as-yet-unidentified mitochondrial iron transporter may be involved in iron influx.

### 2.3 Mitochondria-lipid droplet contacts

Lipid droplets are lipid storage organelles consisting of a triacylglycerol (TAG) and cholesterol ester neutral lipid core surrounded by a phospholipid monolayer studded with proteins to carry out structural, regulatory and enzymatic functions. Lipid droplets are emerging as a critical node in cell metabolism through their actions as both energy-rich fuel reservoirs for cells to tap into during nutrient depletion conditions, as well as a warehouse for lipid materials for use during cell growth and membrane expansion. Mitochondria also are central players in cell metabolism, housing the chemical reactions involved in β-oxidation, the citric acid cycle and oxidative phosphorylation. In addition to these catabolic functions, mitochondria are also involved in anabolic processes as many citric acid cycle intermediates serve as substrates for biosynthetic processes including fatty acid, sterol, amino acid and nucleic acid production. Given the central role of both lipid droplets and mitochondria in metabolism, and specifically in metabolism related to lipids, it makes sense for a highly coordinated system of communication to exist between these organelles.

#### 2.3.1 Mitochondria-lipid droplet contacts promote lipid metabolism

Under nutrient depletion growth conditions (e.g. starvation or hibernation), where carbohydrate availability is reduced, cellular metabolism is remodeled to enhance the use of stored fatty acids for fuel [123]. This remodeling involves the mobilization of fatty acids stored as TAGs from lipid droplets and their subsequent uptake into mitochondria where they are metabolised for ATP production. Membrane contact between lipid droplets and mitochondria has been proposed as an ideal mechanism to facilitate efficient and direct exchange of fatty acids between lipid droplets and mitochondria for metabolism while preventing release of toxic free fatty acids into the cytosol.

Fatty acids, initially localised to lipid droplets under nutrient-rich conditions, are observed to redistribute to mitochondria following nutrient depletion [124,125]. The transport of lipids between these two organelles is thought to occur at mitochondria-lipid droplet contact sites due to the increase in these MCSs during starvation [6124]. While the tethering and lipid transfer machinery involved in this process are not well understood, the SNARE protein SNAP23 has been implicated in mediating mitochondria-lipid droplet contact related to lipid metabolism (Table 1). Knockdown of SNAP23 resulted in decreased mitochondria-lipid droplet contact as well as a decrease in β-oxidation of a radiolabeled fatty acid substrate, suggesting mitochondria-lipid droplet contacts promote mitochondrial β-oxidation under conditions promoting lipolysis [126]. However, the mechanism of how SNAP23 mediates mitochondria-lipid droplet contact, such as if SNAP23 itself acts directly as a lipid droplet-mitochondria tether, has not been determined. Furthermore, while a specific signalling pathway linking the cell's nutrient status to changes in mitochondria-lipid droplet contact has not been well characterised, AMPK, a critical sensor of cellular energy status activated upon nutrient depletion to promote metabolic functions such as β-oxidation and lipolysis, has been implicated in regulating mitochondria-lipid droplet contact [124] (Table 1).

#### 2.3.2 Mitochondria-lipid droplet contacts promote lipid droplet expansion and biogenesis

Less intuitive is the potential role of mitochondria-lipid droplet contact in lipid droplet expansion and biogenesis. While the mechanics of lipid droplet biogenesis are still being elucidated, the prevailing hypothesis is that neutral lipids (such as TAG and sterol-esters), produced by ER resident enzymes, concentrate within the leaflet of the ER phospholipid bilayer before budding from the ER as a nascent lipid droplet [127]. Lipid droplets are then able to grow through either droplet-droplet fusion or acquisition of TAG from the ER through ER-lipid droplet contacts. Additionally, some evidence suggests that lipid droplets are also capable of locally synthesizing TAG independent of the ER [128]. The potential involvement of other organelles in this process is still unclear, however recent work has implicated the mitochondria in lipid droplet expansion and biogenesis [[129], [130], [131]].

Mitochondria isolated from the lipid droplet-associated fraction of brown adipose tissue (BAT), a key site of fatty acid storage and thus lipid droplet biogenesis, are shown to have a decreased capacity for fatty acid oxidation compared to non-lipid droplet-associated mitochondria [129], in contrast to lipid droplet-associated mitochondria in lipid-metabolising tissues [126]. Additionally, stimulation of fatty acid oxidation through cold exposure in BAT results in decreased mitochondria-lipid droplet contact [129], suggesting that mitochondria-lipid droplet contact does not contribute to lipid oxidation in BAT. Additionally, in white adipose tissue (WAT), mitochondrial mass has been found to be significantly higher in differentiating adipocytes than in mature adipocytes [132]. As WAT differentiation involves the rapid accumulation of lipids and production of lipid droplets, the correlation of increased mitochondrial mass during this time suggests mitochondria contribute to lipid droplet biogenesis. Further evidence supporting the role of mitochondria-lipid droplet contact in lipid droplet expansion comes from work identifying specific mitochondria-lipid droplet tethers.

DGAT2, the enzyme responsible for catalyzing the final step in TAG synthesis, is required for lipid droplet biogenesis and expansion. DGAT2 is seen to localize to the ER, where it is specifically enriched in the mitochondria-ER contact site, as well as to lipid droplets following treatment with exogenous lipids [128,133]. DGAT2 localised to lipid droplets interacts with the ER protein FATP1 to facilitate TAG synthesis and lipid droplet expansion [134]. Intriguingly, DGAT2 also possesses a mitochondrial targeting sequence which recruits mitochondria to lipid droplets following treatment with exogenous lipids [133]. This recruitment of mitochondria to lipid droplets by a key enzyme in the TAG synthesis pathway supports the involvement of mitochondria in lipid droplet expansion [130] (Table 1; Fig. 1).

PLIN5, a lipid droplet-associated protein, has also been shown to mediate contact with mitochondria to promote lipid droplet expansion (Table 1; Fig. 1). PLIN5 overexpression has been shown to increase contact between lipid droplets and mitochondria through a 20 amino acid sequence on the C-terminus of PLIN5 [129,135,136]. However, it is not known if this domain interacts with a protein component on the mitochondria, or targets to the mitochondria membrane itself. PLIN5-stimulated mitochondria-lipid droplet contact was found to induce lipid droplet biogenesis, as both the amount of lipid droplets and incorporation of radiolabeled lipids into TAG increased with PLIN5 overexpression [129,135,136]. Conversely, knockout of PLIN5 in mice resulted in a loss of lipid droplets and increased β-oxidation [137]. Importantly, although PLIN5 has also been shown to negatively regulate lipolysis through an inhibitory interaction with the lipase ATGL, this effect of PLIN5 overexpression on lipid droplet accumulation and TAG production was dependent on the mitochondria interacting domain [129,138]. This indicates that the effect of PLIN5 on lipid droplet accumulation is due to its role in mitochondria-lipid droplet contact and not lipolysis inhibition.

The outer mitochondrial membrane protein MIGA2 has also been implicated in facilitating lipid droplet biogenesis through mitochondria-lipid droplet contact [139] (Table 1; Fig. 1). MIGA2 overexpression was shown to increase mitochondria-lipid droplet contact following treatment with exogenous lipids. This MIGA2-mediated mitochondria-lipid droplet contact was dependent on an amphipathic region in MIGA2 that is hypothesized to directly bind the lipid droplet membrane. Additionally, MIGA2 was shown to bind to ER–resident VAPs through a FFAT motif, suggesting that MIGA2 may also mediate mitochondria-ER contact or a triple-contact site between the ER, mitochondria and lipid droplets. MIGA2 knockout adipocytes showed a dramatic decrease in lipid droplet accumulation and TAG production during adipocyte differentiation, as well as a decrease in the size of remaining lipid droplets. Since lipid droplet biogenesis is normally robustly activated during adipocyte differentiation, this suggests MIGA2 is specifically required for the formation of lipid droplets. However, given the ability of MIGA2 to facilitate mitochondrial contact with both lipid droplets and the ER, it is not clear from the evidence if MIGA2 acts directly on lipid droplet biogenesis through mitochondria-lipid droplet contact, or indirectly through mitochondria-ER contact. It would be interesting to assess if either the FFAT motif (ER contact), or the lipid droplet binding domain (lipid droplet contact), or both are necessary to rescue lipid droplet biogenesis in MIGA2 knockout cells.

While the role of mitochondria in lipid droplet metabolism appears well established, what is still unclear is the mechanism by which mitochondria contribute to lipid droplet biogenesis and expansion. One theory is that mitochondrial contact functions to provide energy for ATP-dependent TAG synthesis. This is supported by evidence showing that TAG synthesis is sensitive to oligomycin, an inhibitor of ATP synthase, suggesting that TAG synthesis is dependent on mitochondrial-derived ATP [129]. Additionally, while lipid droplet-associated mitochondria have decreased capacity for β-oxidation under conditions favouring lipid droplet expansion, oxidative capacity for non-fatty acid substrates was actually found to be increased compared to non-lipid droplet-associated mitochondria [129]. This suggests that mitochondria in contact with lipid droplets have elevated ATP production through metabolism of non-lipid fuels.

An alternative hypothesis is that mitochondria contribute to lipid droplet growth by functioning in the synthesis of de novo lipids. In differentiating adipocytes, *de novo* synthesised lipids were shown to be preferentially stored in TAGs over exogenous fatty acids [139]. As the machinery involved in de novo lipogenesis involves factors localised to both the ER and mitochondria, it is reasonable that the spatial proximity of mitochondria to the ER and lipid droplets may act to facilitate synthesis and storage of de novo lipids. Indeed, MIGA2 knockout cells were shown to be unable to incorporate glucose-derived C^14^ into TAG, which was efficiently performed by WT cells [139].

## 3 Peroxisome-organelle interactions and their physiological relevance

**Fig. 2:**
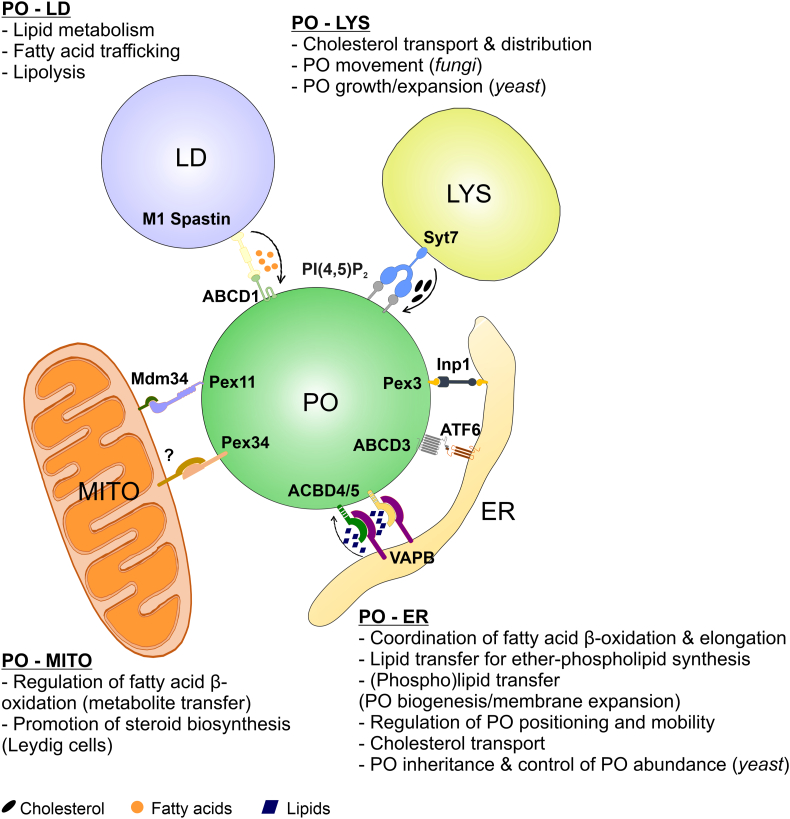
Schematic overview of peroxisome-organelle interactions and their physiological relevance. ABCD1/3, ATP Binding Cassette subfamily D member 1/3; ACBD4/5, Acyl-CoA Binding Domain containing protein 4/5; ATF6α, Activating Transcription Factor 6α; ER, Endoplasmic Reticulum; Inp1, Inheritance of peroxisomes protein 1; LD, Lipid Droplet; LYS, Lysosome; Mdm34, Mitochondrial distribution and morphology protein 34; MITO, Mitochondrion; M1 spastin, isoform M1 of the microtubule-severing protein spastin; Pex, Peroxin; PI(4,5)P2, Phosphatidylinositol-4,5-biphosphate; PO, Peroxisome; Syt7, Synaptogamin-7; VAPB, Vesicle-Associated Membrane Protein (VAMP)-associated Protein B.

Peroxisomes are ubiquitous organelles with major functions in cellular lipid and ROS metabolism. Peroxisomal lipid metabolism requires cooperation and interaction with the ER, mitochondria and lipid droplets [16,140] (Table 1; Fig. 2). Efficient degradation of fatty acids by peroxisomal β-oxidation involves metabolic cooperation with mitochondria and the ER. The synthesis of ether-phospholipids (e.g. plasmalogens enriched in myelin sheaths) and polyunsaturated fatty acids such as docosahexaenoic acid depends on the metabolic interplay between peroxisomes and the ER. Furthermore, peroxisomes are important intracellular signalling platforms modulating physiological and pathological processes including innate immunity, inflammation, and cell fate decision [141].

### 3.1 Peroxisome-ER contacts

Contacts between peroxisomes and the ER have been known about for some time, since peroxisomes were often seen in close association with the ER in early EM images. Indeed, the ER is even thought to contribute to de novo biogenesis of peroxisomes. Although the machinery behind this process is not completely understood [142], it is suggested that the peroxisomal membrane proteins PEX3 and PEX16 may initially be localised in the ER membrane prior to their translocation to peroxisomes, through an ER dependent pathway [143]. In mammalian cells, peroxisome-ER MCSs are mediated by the ACBD4/5-VAP tether (see Section 3.1.1) (Table 1; Fig. 2). The VAP proteins bind to FFAT-like motifs in ACBD4/5 through their MSP (Major Sperm Protein) domain, which facilitates binding to a number of FFAT-containing proteins [144], including PTPIP51 (see Section 2.1.5). The MCSs established between the ER-resident VAP proteins and FFAT-containing domain proteins on other organelles are involved in diverse biological functions, such as lipid transport, calcium homeostasis, signalling regulation, autophagy, and endosome dynamics [145]. Using a proteomic approach, a novel ER-resident protein with a FFAT-binding domain, MOSPD2 (Motile Sperm Domain-containing protein 2), was identified and characterised. This study showed that MOSPD2 binds FFAT-domain containing proteins through the MSP domain, in the same way as the VAP proteins do [145]. While the functions of MOSPD2 are not clear and its effect on MCSs is not understood, it is possible that, like VAP proteins, MOSPD2 also interacts with ACBD4/5, so may represent a novel peroxisome-ER tether [16]. In yeast, the peroxins Pex3, Pex24, Pex32 and the inheritance protein Inp1 have been implicated in peroxisome-ER tethering (see Section 3.1.2) [3,[146], [147], [148]] (Table 1).

#### 3.1.1 Peroxisome-ER contacts facilitate lipid/fatty acid transfer and peroxisome membrane dynamics

In addition to the role of the ER in de novo biogenesis of new peroxisomes, mature peroxisomes also require the ER for their function, which is mediated by MCSs between the two organelles. The acyl-coenzyme A (CoA)-binding domain proteins ACBD5 and ACBD4 act as tethers to mediate peroxisome-ER MCSs, through the binding of their FFAT-like motif to VAP proteins in the ER membrane [[149], [150], [151]]. Both proteins belong to the large ACBD family, whose members are involved in lipid-binding, cellular signalling, lipid metabolic pathways and controlling energy regulation, and are found in eukaryotes and prokaryotes [152]. In addition to their FFAT-like motifs, peroxisomal ACBD4 and ACBD5 possess an acyl-CoA binding domain at the N-terminus (exposed to the cytosol), a coiled-coil region and a C-terminal transmembrane domain. ACBD5 binds very-long-chain acyl-CoAs and is proposed to facilitate their import into peroxisomes for further β-oxidation via the peroxisomal ABC transporter ABCD1. The first patients with a loss of ACBD5 function have recently been identified, suffering from retinal dystrophy and progressive leukodystrophy; ACBD5 deficiency leads to impaired peroxisomal β-oxidation of these very-long chain fatty acids (VLCFAs) and consequent accumulation [153,154]. Many of the metabolic functions of peroxisomes in lipid metabolism are carried out in cooperation with the ER [155]. The ER membrane houses enzymes involved in fatty acid elongation (ELOVLs). It is suggested that the ACBD5-VAP tether contributes to the formation of a peroxisome-ER metabolic hub that allows control of fatty acid chain length (Table 1; Fig. 2). Regulated cooperation at the ER-peroxisome interface can prevent the synthesis of excess amounts of over-long VLCFA through transmission to peroxisomes for degradation via β-oxidation [16]. In support of a peroxisome-ER lipid hub, the long-chain acyl-CoA synthetase ACSL1 was recently identified as a direct interaction partner of ACBD5 and VAPB [156]. Furthermore, peroxisomes and ER cooperate in the synthesis of ether-phospholipids (e.g. myelin sheath lipids), which in mammalian cells is initiated in peroxisomes and completed in the ER. Loss of ACBD5 function resulted in a reduction in ether-phospholipids [157], supporting the notion that the peroxisome-ER contact may facilitate lipid/metabolite transfer for ether-phospholipid synthesis (Table 1; Fig. 2).

Disruption of peroxisome-ER contacts also prevented peroxisomal membrane expansion, which is a pre-requisite for the formation of peroxisomes by membrane growth and division [141,149]. Conversely, overexpression of ACBD5 in various mammalian cell lines increased the interaction between peroxisomes and the ER and induced peroxisomal membrane expansion in a VAP-dependent manner [149,151]. These observations support a role of the ACBD5-VAP peroxisome-ER contact in peroxisome biogenesis and supply of membrane (phospho)lipids (Table 1; Fig. 2). These findings also explain why peroxisomes are hyper-elongated in cells from patients suffering from organelle division defects. Mutations in the shared peroxisome/mitochondria organelle division factors such as DRP1/DNML1 and MFF (see Section 2.1.1) can result in severe disorders with neurological abnormalities and are characterised by defects in the membrane dynamics and division of peroxisomes (and mitochondria) rather than by loss of metabolic functions [[158], [159], [160], [161]]. The hyper-elongation of peroxisomes in MFF-deficient fibroblasts has been suggested to result from a constant ER-peroxisome lipid flow via VAP-ACBD5 MCSs [149,161,162]. As peroxisomes cannot divide due to the loss of functional MFF, lipid supply from the ER causes a pronounced expansion of the peroxisomal membrane. Peroxisome membrane expansion can also be achieved by overexpression of MIRO1, a mitochondrial Rho GTPase, which also targets peroxisomes [162]. MIRO1 functions as a membrane adaptor for microtubule-dependent motor proteins and can exert pulling forces at peroxisomes which promote membrane expansion when peroxisomes are tethered. A comparison of the peroxisome surface area before and after elongation indicates that the globular peroxisome on its own cannot provide sufficient membrane lipids to generate such membrane protrusion; these findings further support the hypothesis that membrane lipids are supplied by the ER through MCSs [162].

Disruption of the peroxisome-ER contact in mammalian cells increased the movement of peroxisomes, suggesting a new role of ACBD5-VAP tethering in the regulation of peroxisome mobility and positioning [149,151] (Table 1; Fig. 2). Furthermore, MIRO1-mediated pulling forces were able to divide and proliferate peroxisomes in fibroblasts due to peroxisome-ER tethering (which prevented movement of peroxisomes) [162]. In mouse hippocampal primary cultures, peroxisomal long range movements were largely diminished and peroxisome number reduced following ACBD5 overexpression, coupled with a redistribution of peroxisomes from the soma to neurites. However, these alterations were independent of VAPB, which might suggest another ACBD5-binding protein contributes to peroxisome-ER contact site formation in neuronal cells [163].

Similar observations have recently been made in the yeast *Hansenula polymorpha* [146]. This study revealed that the peroxins Pex24 and Pex32, which localize to the ER, function as tethers to mediate peroxisome-ER contacts (Table 1). Pex24 and Pex32 belong to the Pex23 protein family, whose members localize to the ER and contain a dysferlin domain. Deletion of Pex24 or Pex32 resulted in a disruption of peroxisome-ER contacts and impaired peroxisome biogenesis, proliferation, positioning at the cell cortex and proper segregation to mother cells and buds (see Section 3.1.2). These defects were suppressed upon introduction of an artificial peroxisome-ER tether. It was also suggested that these proteins may contribute to lipid supply and peroxisomal membrane expansion. Interestingly, accumulation of Pex32 at peroxisome-ER contacts was lost in the absence of the peroxisomal membrane protein Pex11 (Table 1). Additionally, peroxisome-ER contacts were disrupted, indicating that Pex11 functions together with Pex23 family proteins to associate peroxisomes to the ER [146]. *S*. *cerevisiae* Pex11 is also a component of a peroxisome-mitochondrion MCS, indicating that Pex11 may contribute to the formation of different MCSs [164] (see Section 3.3).

#### 3.1.2 Peroxisome-ER contacts ensure accurate peroxisome inheritance

When a eukaryotic cell divides, for example during yeast cell budding, it must partition its organelles between the two daughter cells so both are functionally competent. In yeast, the ER-peroxisome tether is required for peroxisome inheritance during cell division [3]. Knoblach *et al.* showed that in *S*. *cerevisiae*, Pex3, a protein required for peroxisome biogenesis, acts as a receptor for the inheritance factor Inp1, which is localised at the cortical ER. Recruitment of Inp1 to peroxisome-localised Pex3 is required to dock peroxisomes to the cortical ER [147] (Table 1; Fig. 2). This immobilization of peroxisomes at the cell cortex ensures the maintenance of peroxisome populations and a balanced distribution between mother and daughter cells after budding, and may also be involved in the control of peroxisome abundance. Pulling forces exerted by the actin-based class V myosin motor Myo2, and constriction forces exerted by the peroxisomal division machinery, lead to elongation, constriction and division of the peroxisome. The process is asymmetric and leads to the release of larger and smaller peroxisomal fragments, which contain the additional factor Inp2, the peroxisomal adaptor for Myo2, and are transported to the bud. After its release from Myo2, the bud-localised peroxisome can attach to a tether that is recruited by peroxisomal Pex3 binding to Inp1. These studies contributed to the understanding of how MCSs influence the mechanistic processes of cell division and organelle segregation, and how a uniform peroxisome number is maintained in a growing population [147,148].

#### 3.1.3 Peroxisome-ER contacts and stress response

A recent study has demonstrated an interaction between the ER-resident stress sensor ATF6α and the peroxisomal fatty acid transporter ABCD3/PMP70, following treatment with the small molecule Ceapin. Ceapin selectively blocks the protective activity of ATF6α, excluding it from ER exit sites during ER stress, leading to cell death [165,166]. Proteomic analysis identified the peroxisomal transmembrane protein ABCD3/PMP70 as a molecular target of Ceapin. It is suggested that Ceapin induces interactions between ATF6α and ABCD3, tethering the ER and peroxisome and causing ABCD3 to sequester ATF6α from its normal trafficking route without interfering with ABCD3's normal function (Table 1; Fig. 2). These findings present a step towards the understanding of the roles of MCSs in regulating the cellular stress response, as well as the therapeutic potential of modulating the proteostasis network [167].

#### 3.1.4 Peroxisome-ER contacts may facilitate pathogen infection

Peroxisomes have a crucial role in the cellular defence response to infection [168]. However, new studies have suggested that several peroxisomal proteins might also have a role in the pathogen replication cycle, which allows the pathogen to spread in the host. One example used three-dimensional fluorescence microscopy to reveal that peroxisomes surround the inclusion bodies formed by invading *Chlamydia*, where they are located close to the bacteria. Why peroxisomes are necessary for chlamydial infection is still unclear, however peroxisomes may shape the cellular lipid content to produce bacteria-specific plasmalogens [169]. It was also suggested that peroxisomal plasmalogen synthesis is important for the replication of Zika virus (ZIKV) during viral infection, as well as having a crucial role in antiviral defence [170]. Through a BioID assay, a set of ER-peroxisome MCS proteins required for lipid transfer (including the proteins ABCD3, ACBD5, VAPB and VAPA) were identified as exhibiting high confidence interactions with ZIKV proteins [171]. This suggests that viruses may exploit their host lipid synthesis, in particular ether lipids, which are synthesised by peroxisome-ER cooperation (see Section 3.1.1). The peroxisome-ER contact sites might therefore have a role in the synthesis of virus-incorporated lipids via transfer of lipid intermediates [149,151] (Table 1). This lipid synthesis is crucial for virus replication; indeed, decreased peroxisome-specific ether lipid synthesis impairs influenza virus replication [172].

### 3.2 Peroxisome-lipid droplet contacts

As key lipid-metabolising organelles, there needs to be extensive cross-talk between peroxisomes and lipid droplets to regulate organelle function on both sides. The close relationship between peroxisomes and lipid droplets might come from their shared sites of biogenesis. A recent study in yeast has shown that the discrete subdomains of Pex30 on the ER might be sites for nascent lipid droplet and pre-peroxisomal vesicle (PPV) formation. When there is an excess of oleic acid or Pex30, peroxisome number increases and Pex30 localizes to PPVs/peroxisomes, which suggests that the Pex30 subdomains might be the site for PPV formation [173,174]. The same was also observed for lipid droplets. It was also shown that PPVs were associated with lipid droplets at Pex30 subdomains and that the absence of Pex30 causes small and clustered PPVs and lipid droplets, which together suggests these two organelles can form at the same ER site [175,176].

Peroxisome-lipid droplet MCSs have been observed in yeast, mammalian and plant cells [6,7,177]. In COS-7 cells, ~10% of lipid droplets are in contact with peroxisomes at any one time [6]. Since lipid droplets are bounded by a phospholipid monolayer studded with cytosol-exposed proteins, tether proteins on juxtaposed organelles could either bind directly to the lipid droplet membrane via lipid-interacting domains (for example, the ER resident protein DGAT2, which binds the lipid droplet bilayer directly via its C-terminal domain), or via protein-protein interactions between the apposed membranes [26]. Information about the molecular identities of lipid droplet-peroxisome tethers is scarce, with the protein-protein interaction between the lipid droplet membrane-bound AAA ATPase M1 Spastin and the peroxisomal fatty acid transporter ABCD1 being the best characterised [178] (Table 1; Fig. 2).

#### 3.2.1 Peroxisome-lipid droplet contacts regulate fatty acid trafficking and lipid metabolism

As peroxisomes are solely responsible for β-oxidation in yeast and plants, neutral lipids stored in lipid droplets must be transferred to peroxisomes in the form of free fatty acids for oxidative breakdown into acetyl-CoA [179], which requires close coordination between these organelles [180]. When yeast cells are grown in oleic acid as the sole carbon source, which is stored in lipid droplets after uptake, the number of lipid droplet-peroxisome contacts increases, as the oleate must be transferred to peroxisomes to be metabolised by β-oxidation in order to be used to generate energy [179]. Conversely, in mammalian cells,where both mitochondria and peroxisomes cooperate in β-oxidation, excess oleic acid (preferentially oxidised in mitochondria in mammals) actually reduces the number of lipid droplet-peroxisome contacts, instead increasing lipid droplet-lysosome contacts to degrade excess lipid droplets [6].

Ultrastructural studies in yeast showed that peroxisomes and lipid droplets can interact through peroxisomal extensions called pexopodia that extend into lipid droplets. This is proposed to proceed via hemifusion between the lipid droplet monolayer membrane and the outer leaflet of the peroxisome bilayer membrane – this model would imply direct contact between the inner peroxisomal leaflet and the core of the lipid droplet, which would allow the easy diffusion of fatty acids across the monolayer [179]. In mammalian cells, on the other hand, a protein-protein tether established between the lipid droplet protein M1 Spastin and the peroxisomal fatty acid transporter ABCD1 regulates the trafficking of fatty acids between the two organelles (Table 1; Fig. 2). Moreover, M1 Spastin recruits ESCRT III proteins to remodel the lipid droplet membrane, facilitating fatty acid trafficking at these MCSs [178]. Interestingly, cumyl-OOH treatment, which induces lipid peroxidation and consequently oxidative stress, leads to an increase in the ABCD1-mediated contacts between lipid droplets and peroxisomes which might suggest an additional role of this peroxisome-lipid droplet MCS in redox homeostasis [178] (Table 1; Fig. 2). Mutations in the gene encoding Spastin (the most common cause of hereditary spastic paraplegia) cause aberrant fatty acid metabolism in lipid droplets, along with impaired peroxisome movement and distribution and increased lipid peroxidation. This suggests that these disorders may be caused by a defect in fatty acid trafficking between lipid droplets and peroxisomes. In fact, a mutation in M1 Spastin which failed to induce lipid droplet-peroxisome contact formation also impaired fatty acid transport, which corroborates the physiological need for a tether between peroxisomes and lipid droplets [3,178].

Functionally, peroxisome-lipid droplet contacts are also important for efficient lipolysis in response to nutrient deprivation. In *C*. *elegans*, 12 h fasting causes transport of peroxisomes to lipid droplets, via the microtubule-dependent motor protein KIFC3, a process which is required for effective release of lipids from lipid droplets [181]. Mechanistically, the peroxisomal protein PEX5 in mammalian adipocytes (orthologous to the lipolysis-promoting PRX-5 in *C*. *elegans*) chaperones the translocation of the lipase ATGL from the cytoplasm to the lipid droplets at peroxisome-lipid droplet contacts, allowing the liberation of stored triglycerides as metabolic substrates during nutrient deprivation, suggesting a role for peroxisome-lipid droplet contacts in the utilisation of lipids stored in lipid droplets under a variety of cellular conditions.

### 3.3 Peroxisome-mitochondria contacts

Peroxisomes and mitochondria are highly complementary organelles, working in concert to execute a number of key cellular functions, such as metabolic processes (including β-oxidation of fatty acids), redox/ROS homeostasis and anti-viral signalling [182]. Indeed, peroxisomes and mitochondria are so closely connected that the biogenesis of the two organelles involves a number of shared proteins, leading to a degree of co-regulation in the number and dynamics of these organelles [183]. It is increasingly becoming clear that a physical peroxisome-mitochondria connection, at MCSs, can facilitate this well-reported functional peroxisome-mitochondria interplay, allowing for coordinated signalling and metabolite exchange between the two compartments [16] (Table 1; Fig. 2).

In yeast, where β-oxidation of fatty acids is carried out solely in peroxisomes, metabolic communication between peroxisomes and mitochondria is especially vital for the utilisation of fatty acids as an energy source. β-oxidation in yeast generates acetyl-CoAs, which must be transferred as membrane-permeable intermediates to mitochondria in order to be used for ATP production by oxidative phosphorylation, while reducing equivalents (malate and 2-oxoglutarate) can be shuttled between peroxisomes and mitochondria as one method of regenerating NAD^+^ in the peroxisomes for subsequent rounds of β-oxidation [[184], [185], [186]]. Even in mammals, where both peroxisomes and mitochondria house enzymes for β-oxidation, there is a similar need for bidirectional peroxisome-mitochondria metabolite transfer. As peroxisomes can only chain-shorten fatty acids, intermediates are shuttled to mitochondria in the form of acylcarnitine esters or free acids to ensure full oxidation [140]. These coordinated metabolic processes also produce ROS, contributing to the closely inter-related redox homeostasis and signalling between the two organelles, though the exact messengers remain unclear [187].

The initial observation that around 50% of peroxisomes in yeast cells were found adjacent to mitochondria at subdomains of acetyl-CoA synthesis (marked by the presence of pyruvate dehydrogenase [PDH]) suggested that close, spatially regulated juxtaposition of the two organelles could be important for their interrelated metabolic functions [188]. Both of these organelles also form important functional connections with the ER, with peroxisome-mitochondrial colocalisation often coinciding with ERMES in yeast (see Section 2.1). The peroxisomal membrane protein Pex11 has been shown to interact with the mitochondrial outer membrane ERMES component Mdm34 in *S*. *cerevisiae*, with the observed decrease in the proportion of peroxisomes adjacent to ERMES in *Δpex11* cells leading to the proposal that this Pex11-Mdm34 interaction could act as a peroxisome-mitochondria tether in yeast [164] (Table 1; Fig. 2). However, similar to known tethers at other MCSs, peroxisome-mitochondria contacts were not completely abolished in *Δpex11* cells, suggesting there may be numerous tethering molecules between these two organelles.

Peroxisome-mitochondria contact sites have also been observed in mammalian cells [189], though are not as well characterised on a molecular level as those in yeast. Estimates from multispectral analysis of 6 tagged organelles imaged simultaneously in live COS-7 cells suggested around 20% of peroxisomes are in contact with mitochondria at any one time [6]. Interestingly, peroxisome-mitochondria contacts are facilitated by microtubules in these cells, since the number of contacts was reduced upon treatment with the microtubule depolymerising agent nocodazole (in contrast with peroxisome-ER contacts which were unaffected) [6]. Peroxisome-mitochondria contacts seem to preferentially occur at elongated peroxisomal membrane protrusions [190], which is reminiscent of the peroxisome tubules that are observed to contact mitochondria [191] and lipid droplets [192] in *Arabidopsis*, though it is currently unclear if these represent conventional MCSs or a more dynamic, transient form of organelle communication. Juxtaposition of peroxisomes and mitochondria has also been observed in neuronal cells [163], but at a much higher frequency than in COS-7 cells (~80% of peroxisomes apposed to mitochondria), which may indicate cell type-specific differences in the extent of MCSs. It is also interesting to consider the high level of peroxisome-mitochondria contact in neuronal cells, given the low level of β-oxidation that occurs in the brain, as it suggests that mitochondria-peroxisome contacts may function in multiple cellular processes in addition to β-oxidation.

#### 3.3.1 Peroxisome-mitochondria contacts regulate fatty acid β-oxidation

Recently, a systematic study investigating all combinations of inter-organelle contact sites has further characterised the molecular basis of the peroxisome-mitochondria interaction in yeast. Here, split fluorescent proteins fused to the cytoplasmic side of various resident peroxisomal/mitochondrial membrane proteins were used to visualise sites where peroxisome and mitochondria were close enough for bimolecular fluorescence complementation to occur [7]. Importantly, the peroxisome-mitochondria contacts detected by this reporter method localised to peroxisome-mitochondria interfaces and recapitulated previous observations, such as peroxisome residing adjacent to ERMES sites and PDH subdomains within mitochondria, suggesting it could accurately report on physiological contact sites.

By using a variety of different lengths of cytoplasmic linker in the reporter fusions, the peroxisome-mitochondria contact distance was estimated to be 10–80 nm, consistent with reports for other MCSs. To identify candidate tether proteins, a high-throughput overexpression screen was performed, highlighting 12 peroxisomal or mitochondrial proteins that, when overexpressed, caused an expansion of the peroxisome-mitochondria contact reporter signal [7]. The authors focussed on the mitochondrial fusion protein Fzo1 and the peroxisomal membrane protein Pex34 (a distant Pex11 homologue [7]) as novel tether proteins, demonstrating that both proteins, when individually overexpressed, were enriched at MCS and specifically increased the extent of peroxisome-mitochondria contacts (Table 1). Interestingly, overexpression of these putative tethers also reduced peroxisome motility, which may be a relevant physiological function or simply a side-effect of artificially increased organelle tethering. Crucially, this study also identified a physiological role for peroxisome-mitochondria tethering in the regulation of fatty acid β-oxidation (Table 1; Fig. 2). When yeast were grown on oleate as their sole carbon source (conditions in which β-oxidation becomes essential for energy generation), the number of peroxisome-mitochondria contact sites detected by fluorescent reporters increased. Concerted mitochondria-peroxisome metabolism was assayed by supplementing cells with radiolabeled octanoate (C8:0), which in yeast must be metabolised by β-oxidation in peroxisomes to generate acetyl-CoA, before being subsequently degraded in mitochondria via the TCA cycle to CO_2_ and H_2_O. Overexpression of Pex34 led to increased radiolabelled CO_2_ release, suggesting the peroxisome-mitochondria tether facilitates the transfer of acetyl-CoA between the two organelles for efficient fatty acid degradation and energy generation. Notably, overexpression of Fzo1 did not lead to this increase in β-oxidation, implying it may be part of an independent peroxisome-mitochondria tether complex with a different, as yet undiscovered, function [7].

#### 3.3.2 Peroxisome-mitochondria contacts promote steroid biosynthesis

In testosterone-producing Leydig cells, a molecular ‘tug-of-war’ between peroxisomes and mitochondria, mediated by simultaneous trafficking of the dual-localised acyl-CoA binding protein ACBD2 to both of the organelles, has been proposed as a molecular mechanism drawing peroxisomes and mitochondria into close proximity [193] (Table 1). Functionally, these connections promote steroid biosynthesis in a manner dependent on the acyl-CoA binding ability of ACBD2. Moreover, colocalisation of the two organelles was increased upon cAMP stimulation, suggesting regulation of these contact sites is important for tightly-controlled, responsive metabolism. In agreement with the data obtained in yeast, this supports a physiological role for peroxisome-mitochondrial contacts in coordinating the complementary metabolic processes between the two organelles, but it remains to be seen whether this involves the direct shuttling of key intermediates between the two compartments, which is technically challenging to observe.

#### 3.3.3 Peroxisome-mitochondria contacts may regulate mitochondrial redox homeostasis

Another class of metabolites that could potentially be exchanged at sites of peroxisome-mitochondria contact are ROS. While both peroxisomes and mitochondria are major producers of ROS in the cell, and have therefore historically been assumed to have highly oxidizing environments, the luminal environment of the peroxisome has been shown to be reducing in comparison to the cytoplasm, potentially due to the high proportion of peroxisome-localised antioxidants such as catalase [194]. While this reducing capacity likely acts to quench peroxisome-derived ROS, evidence suggests that peroxisomes may also function to quench ROS from non-peroxisomal sources, including mitochondria.

In models of peroxisome biogenesis disorders (PBDs), where cells lack functional peroxisomes, mitochondria exhibit phenotypes characteristic of mitochondrial oxidative stress including structural abnormalities, respiratory chain dysfunction, loss of membrane potential and increased expression of the superoxide dismutase SOD2 [[195], [196], [197], [198]]. This mitochondrial dysfunction can be rescued by antioxidant treatment, indicating that oxidative stress contributes to mitochondrial dysfunction in PBD cells [197]. Additionally, in cells with functional peroxisomes, mitochondrial redox homeostasis has been shown to be sensitive to the reducing capacity of peroxisomes specifically. Mitochondrial ROS was shown to be elevated when catalase was knocked out or chemically inhibited, and restoring targeting of catalase to peroxisomes also rescued the loss of mitochondrial membrane potential observed in late-passage cells [[199], [200], [201]]. Similarly, compromising peroxisome redox capacity using a peroxisome-targeted photosensitizer to overload peroxisomes with peroxisome-generated ROS was shown to increase mitochondrial oxidative state [199]. Together, these results indicate that mitochondrial redox homeostasis is sensitive to the reducing capacity of peroxisomes and suggest that peroxisomes may function as a sink for mitochondria-derived ROS.

The non-specificity and high reactivity of ROS means that they are short lived species, unable to travel long distances. To avoid oxidative damage by reaction with non-intended proteins and lipids, ROS production is spatially linked to reducing components. If peroxisomes are one of these reducing components, it stands to reason that they would need to be in spatial proximity of mitochondria, such as the close proximity provided by MCSs, to receive mitochondrial ROS within the lifetime of the ROS. While exchange of ROS at sites of peroxisome-mitochondria membrane contact has yet to be shown, ROS exchange has been shown to occur at contact sites between the ER and mitochondria, indicating ROS exchange at MCSs is feasible [202].

### 3.4 Peroxisome-lysosome/vacuole/endosome contacts

Similarly to mitochondria, the most obvious contact between peroxisomes and lysosomes/vacuoles occurs during the degradation of damaged or unwanted peroxisomes and subsequent recycling of organelle components. The interplay between peroxisomes and autophagosomes has recently been reviewed [203]. However, novel physiological functions of MCSs, independent of degradation, have been elucidated. Although numerous proteins have been implicated in the formation/regulation of peroxisome-lysosome contacts [204,205], few have been directly shown to have a tethering function. The distinct protein and membrane phosphoinositide profiles of different organelles and endolysosomal compartments allows MCS specificity via restricted tether formation, exemplified by the sole characterised lysosomal-peroxisomal tether formed by the lysosomal membrane protein Syt7 binding to PI(4,5)P_2_, which is enriched in the peroxisomal membrane, via its C2AB domain [205] (Table 1; Fig. 2).

#### 3.4.1 Peroxisome-lysosome contacts mediate cholesterol transport and VLCFA metabolism

In mammalian cells, as previously discussed (see Section 2.2.2), cholesterol is primarily taken up from the external environment by endocytosis in the form of LDL. This is subsequently trafficked via the endocytic pathway to lysosomes for processing, before being distributed to downstream compartments, including the plasma membrane, where it contributes to membrane structure, metabolism and intracellular signalling [206].

The importance of peroxisomes in intracellular cholesterol trafficking was first identified via an unbiased RNAi screen for proteins regulating cholesterol transport out of lysosomes, as the pool of candidate knockdown genes causing a defective cholesterol trafficking phenotype was significantly enriched in peroxisomal genes [205]. Peroxisomal and lysosomal markers colocalised at dynamic and transient contact sites between the organelles, which were reduced if key peroxisome, lysosome or cholesterol trafficking proteins were depleted. Peroxisome-lysosome contacts are at least in part bridged by the lysosomal protein Syt7 binding to PI(4,5)P_2_ in the peroxisomal membrane, with both components being required for the transport of cholesterol from lysosomes to peroxisomes (Table 1; Fig. 2). Importantly, the extent of these peroxisome-lysosome contacts was reversibly reduced following cholesterol depletion, indicating physiological regulation by cellular cholesterol status. Peroxisome-ER contacts have also been implicated in the trafficking of cholesterol out of the lysosome [207], suggesting a novel, non-vesicular mechanism by which peroxisomes facilitate the transport of LDL-derived cholesterol from the lysosome to the ER by direct contacts with both organelles, perhaps even acting as a transient carrier to buffer cellular cholesterol levels and/or shuttle it to downstream compartments as required.

Highlighting the importance and clinical relevance of peroxisomes and peroxisome-lysosome contacts in normal cholesterol distribution, patients with peroxisomal disorders display intracellular cholesterol accumulation resembling patients with direct cholesterol transport disorders. Defects in intracellular cholesterol distribution perturb normal cell and membrane function, for example a recent study has associated a reduction in peroxisome-lysosome mediated cholesterol transport with impaired ciliogenesis [204]. Maharjan et al. identified TMEM135 (PMP52) as a novel peroxisomal protein regulating peroxisome-lysosome contacts. Knockdown of TMEM135 in mammalian cells decreased peroxisome-lysosome colocalization (without changing organelle numbers) and increased cholesterol accumulation in the lysosome, suggesting these TMEM135-mediated contacts are required for transport of internalised cholesterol from the lysosome to peroxisomes (Table 1). Interestingly, TMEM135 depletion also impaired cilia formation by reducing trafficking of the ciliary vesical GTPase Rab8 to centrioles, which could be rescued by cholesterol supplementation [204]. Together, this suggests a model by which TMEM135-dependent peroxisome-lysosome contacts are necessary for maintaining the physiological distribution of intracellular cholesterol, which in turn is essential for ciliogenesis. Additionally, peroxisomal dysfunction caused by Schwann cell-specific PEX5 knockout leads to secondary lysosomal storage disorder-like phenotypes and subsequent peripheral neuropathy in mice, due to an accumulation of gangliosides as lysosome-generated VLCFAs cannot be degraded in peroxisomes [208]. Altogether, this suggests a role for peroxisome-lysosome contacts in cholesterol transport and VLCFA homeostasis.

#### 3.4.2 Peroxisome-vacuole/endosome contacts promote peroxisome expansion and distribution

Recently, a novel peroxisome-vacuole contact site has been described in the yeast *H*. *polymorpha* [209], distinct from the transient contact preceding fusion of the two organelles leading to peroxisome degradation (micropexophagy) [210]. Several systematic studies have also detected this contact in *S*. *cerevisiae* using split fluorescent protein reporters [7,211]. Notably, in *H*. *polymorpha*, no peroxisome-vacuole contacts were detected by EM when the cells were grown on glucose-containing media, which represses peroxisome growth (cells in these conditions typically only possess one small peroxisome), however, upon switching to methanol-containing media, which promotes rapid peroxisome expansion and development, these MCSs could be observed [212]. These peroxisome-vacuole contacts were dependent on the peroxisomal membrane protein Pex3, which accumulated at the peroxisome-vacuole interface upon induction of peroxisome growth, and was sufficient to form peroxisome-vacuole MCSs under peroxisome-repressive conditions when overexpressed, though the molecular mechanism underlying this remains to be elucidated (Table 1). The presence of peroxisome-vacuole contacts exclusively under peroxisome growth conditions raises the possibility that the vacuole could be acting to provide a source of lipids to fuel the rapid expansion of the peroxisome membrane, similar to the lipid transfer that occurs at peroxisome-ER MCS [149] (see Section 3.1.1), however, this has yet to be proven.

MCSs are also crucial for the transport and positioning of many organelles [3]. For example, in filamentous fungi, peroxisomes hitchhike on endosomes for long-range movement along microtubules, via the interaction of peroxisomes with the endosome-associated protein PxdA, generating MCSs that allow the peroxisomes to hitch a ride on endosomes as they are moved by the microtubule-based motors dynein 1 and kinesins [213,214] (Table 1).

## 4 Golgi-organelle interactions and their physiological relevance

**Fig. 3:**
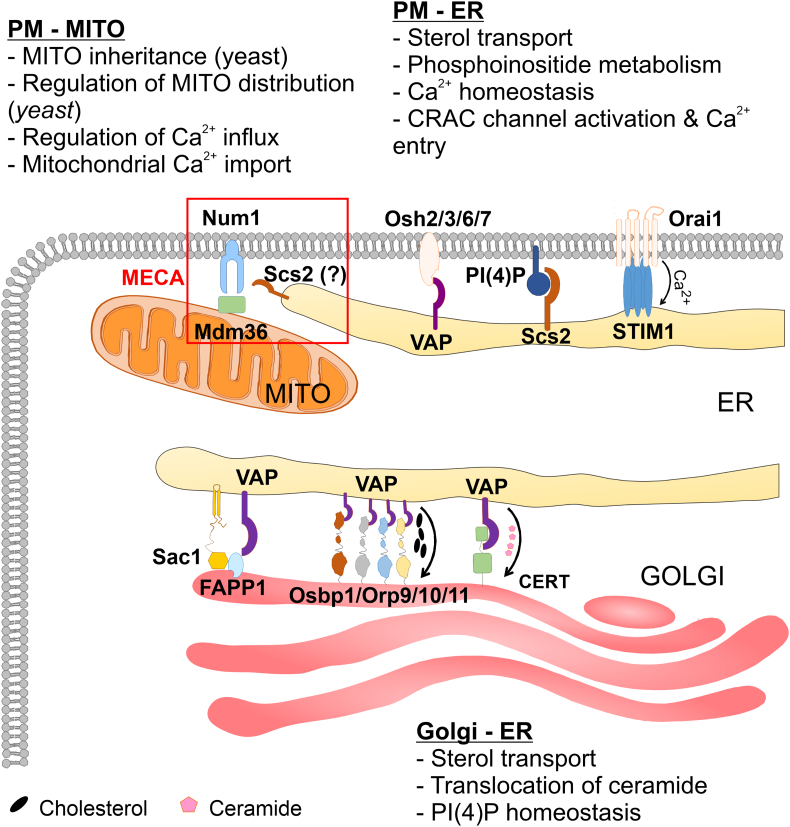
Schematic overview of Golgi-ER and plasma membrane-organelle interactions and their physiological relevance. CERT, Ceramide Transport Protein; FAPP1, Phosphatadyl-four-phosphate-adaptor protein 1; ER, Endoplasmic Reticulum; GOLGI, Golgi Apparatus; Mdm36, Mitochondrial distribution and morphology protein 36; MECA, Mitochondria-ER cortex anchor; MITO, Mitochondrion; Num1, Nuclear migration protein 1; Orai1, Calcium release-activated calcium channel protein 1; Orp9/10/11, Oxysterol-binding protein related protein 9/10/11; Osbp1, Oxysterol-binding protein 1; Osh2/3/6/7, Oxysterol-binding protein homology 2/3/6/7; PI(4)P, Phosphatidylinositol-4-phosphate; Sac1, suppressor of actin 1; Scs2, suppressor of chromosome segregation protein 1 (CSE1) 2; STIM1, Stromal Interaction Molecule 1; VAP, Vesicle-Associated Membrane Protein (VAMP)-associated Protein.

As a major trafficking and protein-sorting hub of the cell, the Golgi complex needs to communicate with a range of organelles to ensure processed cargo is directed to its correct destination [215]. Systematic analysis of the whole-cell organelle interactome in mammalian COS-7 cells has revealed that the Golgi makes a relatively small number of contacts with the ER, mitochondria, peroxisomes, lysosomes and lipid droplets [6], and the Golgi also forms three-way interactions with the ER and lipid droplets in hepatocytes following Hepatitis C infection [216]. However, only Golgi-ER MCSs have been characterised on the molecular and functional level [20] (Table 1; Fig. 3).

### 4.1 Golgi-ER contacts

There is extensive bidirectional communication between the Golgi and the ER, due to their shared function ensuring accurate biosynthesis and distribution of essential intracellular and secreted components. Newly synthesised proteins and lipids are trafficked from the ER to the cis face of the Golgi for further processing and sorting, before being released to their final destination from the trans-Golgi network (TGN) [215]. While vesicle-mediated transport of these proteins and lipids between the Golgi and the ER is well characterised, it is becoming increasingly clear that non-vesicular transfer via MCSs also plays a key role [20] – indeed, non-vesicular transport of ceramide can compensate when vesicular trafficking pathways are blocked in both yeast and mammalian cells [217,218]. Consequently, several of the identified ER-Golgi tether proteins also possess lipid transfer functions to facilitate this [20] (Table 1; Fig. 3). So far, no integral Golgi proteins have been identified as ER-TGN tethers, however all known cytoplasmic tether components contain PH domains that preferentially bind the phosphoinositide PI(4)P, which is enriched at the TGN membrane [219]. The nature and functions of ER-Golgi MCSs are likely to vary between different organisms, due to large variations in the structure and arrangement of the Golgi across eukaryotes [220]. ER-Golgi MCSs have been extensively reviewed (see [20,215,220,221]), but some of the identified physiological functions will be briefly discussed here.

#### 4.1.1 Golgi-ER contacts facilitate lipid transfer

As previously discussed (see Section 2.1.3), the ER is the major biosynthetic hub for lipids in the cell, some of which need to be subsequently transported to the Golgi for incorporation into the Golgi membrane, distribution around the cell or further processing by Golgi-resident enzymes [221]. In mammals, several members of the oxysterol-binding protein/OSBP-related protein family (ORPs) have been proposed as components of ER-TGN tether complexes that facilitate direct transport of sterols between the two apposing membranes, bypassing earlier secretory compartments [20] (Table 1; Fig. 3). Eight of the twelve human ORP proteins, and three of the seven homologous Osh proteins in *S*. *cerevisiae*, possess FFAT motifs, allowing them to interact with VAP proteins (or their yeast homologues Scs2/Scs22) in the ER membrane at MCSs in a manner inversely proportion to their sterol-binding function [222]. Of these, two – OSPB1 and ORP9 – have been shown by FRET-FLIM to be important as redundant VAP-binding tethers for the formation of ER-TGN contacts, and while ORP10 appears to be important for the maintenance of these contacts, it is unclear whether this is a bona fide tether as it only possesses a putative FFAT motif [219].

Mechanistically, ORP9 localizes to the TGN via a PI(4)P-preferring PH domain, and can efficiently transfer cholesterol between liposomes in vitro [223]. Knockdown of ORP9 results in cholesterol accumulation in the endosomal/lysosomal compartment, suggesting ORP9 acts to transfer endocytosed cholesterol between the ER and TGN under normal conditions, which when blocked overwhelms the lysosomal trafficking pathway instead. Interestingly, ORP9 knockdown also causes Golgi fragmentation, suggesting a role for ER-TGN MCSs and/or normal cholesterol flux in Golgi integrity [223]. OSBP1 forms ER-TGN tethers by a similar mechanism, simultaneously binding PI(4)P and VAPA on separate membranes in vitro and promoting PH- and FFAT-dependent ER-TGN tethering in vivo [224]. Overexpression of WT OSBP1, but not a FFAT mutant, resulted in a reduction in lipid droplet formation from exogenous cholesterol, whereas knockdown or inhibition of OSBP1 led to decreased cholesterol in the TGN but increased cholesterol in lipid droplets [225]. Since lipid droplets form from the ER, this suggests OSBP1 counteracts the retrograde flux of cholesterol into the ER by driving ER to TGN cholesterol transport at ER-TGN MCSs [224]. Altogether, this indicates that ORP9 and OSBP1 not only function as physical tethers at ER-TGN MCSs, but also function to transport cholesterol between the two membranes to maintain normal cholesterol flux.

In mammals, ceramide is another lipid that must be transported between the ER and the Golgi, as it is synthesised in the ER but can only be converted to sphingomyelin, which is a crucial component of axonal myelin sheaths, in the Golgi [218]. The PI(4)P-binding protein CERT was initially identified as the essential Golgi-associated factor mediating the ATP-dependent, non-vesicular ER to Golgi ceramide translocation, and was shown to be able to drive intermembrane ceramide transfer in vitro via a ceramide-binding domain [218]. It was subsequently demonstrated that CERT binds the VAP proteins via its FFAT motif, with its ER-TGN tether function being required for ceramide transfer in cells [226], providing another example of lipid-binding proteins that simultaneously bring the ER and Golgi membranes into close apposition as MCS tethers, while also carrying out a lipid transfer function.

#### 4.1.2 Golgi-ER contacts regulate phosphoinositide distribution and homeostasis

The phosphoinositide composition of the Golgi membrane is highly regulated, with localised enrichment of PI(4)P at the TGN being crucial for the concentration and spatial sorting of certain lipid and protein cargoes [20]. PI(4)P turnover is regulated by ER-TGN MCSs via several mechanisms. Interestingly, the ER to TGN cholesterol transport mediated by OSBP is energetically driven by PI(4)P transfer in the opposite direction. The same lipid transfer domain in OSBP1 can transfer either PI(4)P or cholesterol between membranes in vitro, as long as its tether function is also intact, implying a cycle whereby OSBP1 alternately exchanges cholesterol and PI(4)P between the ER and TGN membranes at ER-TGN MCSs [224] (Table 1; Fig. 3). Once at the ER membrane, PI(4)P can be hydrolysed to PI by the ER-resident phosphatase Sac1 [224], while PI(4)P is regenerated at the TGN by PI(4)-kinases such as PI4KIIIβ, which is recruited to the TGN membrane by the Golgi protein ACBD3 [152,227]. Coupled together, this maintains the PI(4)P concentration gradient between the TGN and ER, both ensuring the directionality of cholesterol transport and establishing the difference in membrane composition that is required for downstream signalling and function [225]. Importantly, since ER-Golgi tethers are established by PH-domain binding to PI(4)P, the balance of PI(4)P transport out of the TGN versus its synthesis may represent a mechanism by which the extent of ER-Golgi tethering may be regulated [221].

While this cholesterol/PI(4)P exchange model suggests that ER-localised Sac1 acts to dephosphorylate PI(4)P after it has been transferred to the ER membrane (in *cis*), an alternative hypothesis has proposed that Sac1 can also act directly on PI(4)P in the TGN membrane across the ER-TGN MCS (in *trans*). This is based on evidence that destabilising ER-TGN MCSs, for example by VAP knock-down, results in PI(4)P accumulation at the Golgi, assuming that closer membrane contacts are required for Sac1 activity in *trans* as opposed to OSBP1-dependent Sac1 activity in *cis* [228]. Phosphatidyl-four-phosphate-adaptor-protein-1 (FAPP1) was identified as a regulator of this Sac1 *trans* activity, as its knockdown resulted in TGN accumulation of PI(4)P without disrupting ER-TGN MCSs [228]. FAPP1 binds PI(4)P and localizes to ER-TGN MCSs, forming a tripartite complex by binding the VAP proteins and Sac1. *In vitro* liposome studies showed that FAPP1 has a strong stimulatory effect on Sac1 dephosphorylation of PI(4)P in *trans*, but a far smaller effect in *cis*, implying FAPP1 acts as an adaptor at closely-associated ER-TGN MCSs to reduce PI(4)P levels in the TGN via direct Sac1 activity across the MCS [228]. Functionally, FAPP1- and Sac1-dependent reduction of PI(4)P at the TGN decreases secretion of certain cargoes from the TGN, suggesting regulation of PI(4)P turnover by modulating ER-TGN MCS complexes fine-tunes protein trafficking through the secretory system [228]. Together, these non-exclusive models raise the possibility that ER-TGN MCSs with different compositions may influence PI(4)P homeostasis in independent ways, perhaps depending on cellular context.

## 5 Plasma membrane-organelle interactions and their physiological relevance

As well as acting as a physical boundary around the cell, the plasma membrane (PM) plays a number of key roles in cellular physiology, including transport/trafficking of ions and molecules between the inside and outside of the cell, relay of extracellular signals and control of morphology/polarity. As a result, the PM must act in concert with other cellular components, so accordingly forms MCSs with numerous organelles (Fig. 3). Interactions between PM-ER and PM-mitochondria are the best characterised [21,229]; novel MCSs between the PM and vacuoles, lipid droplets and peroxisomes have recently been observed but the molecular composition and physiological function of these contacts is currently unknown [7,230].

### 5.1 Plasma membrane-ER contacts

In yeast, ER-PM contacts are frequently observed due to the extensive network of cortical ER at the cell periphery in these cells – in fact, split fluorescent reporters have been used to demonstrate that virtually all of the cortical ER is within 20 nm of the PM and therefore comfortably within the range of MCS formation [231]. Interestingly, several of the reported ER-PM tether proteins are homologues of the mammalian VAP family of proteins, which are implicated in forming MCSs between the ER and numerous other organelles [87,149,151] (Table 1). Since these VAP proteins may participate in a number of MCS complexes simultaneously, how they are regulated independently is an important outstanding question.

#### 5.1.1 Plasma membrane-ER contacts regulate sterol transport

As the ER is a major site of lipid biosynthesis in the cell, a key role of ER-PM contact sites is to carry out the regulated non-vesicular transport of essential lipids from their site of biogenesis to the PM, where they contribute to membrane integrity and function. In particular, oxysterol-binding protein–related proteins (e.g. the Osh family in yeast), have been implicated in the transport of sterols from the ER to the PM [232]. In *S*. *cerevisiae*, 4 members of the Osh family of proteins (Osh2,3,6 and 7) are observed to be localised to ER patches adjacent to the PM – interestingly, while Osh2 and Osh3 contain a FFAT motif predicted to bind ER resident VAP proteins, this does not appear to be essential for this localisation since Osh6 and Osh7 do not possess such a motif [233] (Table 1; Fig. 3). The Osh proteins have multiple membrane binding surfaces, allowing them to simultaneously bind to both the ER and the PM in vivo. Elegant in vitro experiments studying Osh-mediated sterol transport between liposomes, where the donor and acceptor liposomes were either in close proximity or separated with a semipermeable barrier, demonstrated that the two membranes need to be closely apposed for Osh-induced sterol transfer to occur. Together, this suggests a model whereby the Osh proteins physically bridge the ER and PM, to bring them into close enough proximity for sterol transfer between the two membranes to be facilitated [233].

Structural reorganisation of the PM provides a novel mechanism for the regulation of PM-ER contacts. In *S*. *pombe*, PM invaginations mediated by large immobile protein complexes known as eisosomes help to stabilise local PM-ER contacts [234]. Interestingly, these seem to be able to directly restrict cortical ER remodeling to modulate PM-ER contacts, allowing a degree of plasticity to regulate cortical processes depending on cellular demands. Close apposition of cortical ER and morphologically similar PM furrows (caveolae) has also been observed in animal cells [235], raising the possibility that this may represent a conserved mechanism by which PM-ER cross-talk is regulated.

#### 5.1.2 Plasma membrane-ER contacts regulate phosphoinositide metabolism

Osh-mediated tethering of the ER and PM has been proposed to regulate another important function of the PM, phosphoinositide metabolism, which is important for a range of signalling pathways. In yeast cells lacking the Osh proteins, the ER displays a more perinuclear distribution, and the PM contains significantly more PI(4)P [236], modulating PM charge and, consequently changing its electrostatic protein binding properties [237]. Mechanistically, the Osh proteins regulate PM PI(4)P homeostasis by activating the ER-resident PI(4)P phosphatase Sac1 at ER-PM contacts as part of a MCS complex including the VAP homologues Scs2 and Scs22. However, deletion of Scs2 and Scs22 causes a smaller increase in PM PI(4)P levels than deletion of Sac1, suggesting the existence of additional ER-PM tethers in *S*. *cerevisiae* that can compensate. To determine possible additional tether candidates, proteins that bound to both Sac1 and Scs2 were identified by SILAC [238]. Of these, Ist2 and the tricalbin (Tcb) family of proteins also localised to the cortical ER, suggesting they may take part in the ER-PM MCS complex (Table 1; Fig. 3). Supporting this, strains lacking Scs2/22, Ist2 and Tcb1/2/3 showed a drastic reduction in cortical ER, which was instead collapsed in the cytosol, as well as increased PI(4)P in the PM. Interestingly, these cells, in which PM-ER contacts are disrupted, constitutively activated their unfolded protein response (UPR), indicative of ER stress, suggesting that ER-PM contacts are also required for some uncharacterised aspect of normal ER function. Mammalian proteins at MCSs involved in phosphatidylinositol delivery between the ER and the PM include NIR2 and C2CD2L/TMEM24 [239,240].

#### 5.1.3 Plasma membrane-ER contacts in Ca^2+^ homeostasis and autophagy

In mammalian cells, it has been demonstrated that ER-PM contacts play an important role in refilling the ER Ca^2+^ store following its depletion, to maintain cellular Ca^2+^ homeostasis [241]. The ER-localised Ca^2+^ sensor STIM1 redistributes to clusters closely apposed to the PM (within 10–25 nm) following cellular Ca^2+^ depletion with EGTA [242], via a direct interaction with the PM store-operated Ca^2+^ channel Orai1, which it activates [243]. Thus, similarly to mitochondria-PM contacts (see Section 5.2), this ER-PM contact acts to specifically and spatially target Ca^2+^ entry to the required location for repletion of ER Ca^2+^ stores to efficiently maintain cellular Ca^2+^ homeostasis (Table 1; Fig. 3). Recent evidence in plants has also implicated ER-PM contacts in facilitating autophagy, with autophagosome formation being initiated at the interface between the ER, PM and F-actin [244]. The continuous discovery of new functions, such as these, mediated by organelle-PM contacts shows the importance and versatility of these MCSs, which will likely be a burgeoning area of research in the future.

### 5.2 Plasma membrane-mitochondria contacts

While the mechanics of mitochondria-PM contacts have not been determined in mammalian cells, this contact has been well characterised in yeast. The core component of mitochondria-PM contact sites in yeast is Num1, a cortical protein shown to interact with dynein as well as mitochondria fission machinery [245,246]. Num1 directly binds PI(4,5)P_2_ in the PM through a PH domain, as well as cardiolipin in the mitochondria membrane through a coiled coil domain, thereby bridging mitochondria and the PM [[247], [248], [249], [250]] (Table 1; Fig. 3). Num1 also self-associates through the coiled coil domain into clusters which are required for Num1's tethering capacity [247,249]. Mdm36, a soluble protein peripherally recruited to the mitochondria membrane, is also recruited to Num1-mediated mitochondria-PM contact sites through a coiled coil-mediated interaction with Num1, and is proposed to facilitate oligomerization of Num1 into functional clusters [247,249]. The ER also localizes to sites of mitochondria-PM contact. ER proteins are found to co-purify with Num1 and the ER is shown to be present in all cases of mitochondria-PM contact [247]. Num1 has been shown to interact with the ER resident membrane protein Scs2 (homologous to the mammalian VAP proteins), with loss of Scs2 leading to defective Num1 distribution [251,252]. This three-way mitochondria-PM-ER contact site is termed the mitochondria-ER cortex anchor (MECA).

#### 5.2.1 Plasma membrane-mitochondria contacts function in mitochondrial inheritance

Mitochondrial distribution within the cell has profound consequences for the inheritance of these organelles. Contact between mitochondria and the PM has been shown to facilitate correct partitioning of mitochondria between mother and daughter cells in *S*. *cerevisiae*. During budding, mitochondria are anchored to the bud tip as well as the mother cell cortex and are partitioned evenly in both directions by bi-directional actin-dependent movement [253]. Num1 localised to the mother cell cortex acts to retain mitochondria in the mother cell by mediating PM-mitochondria contact. Loss of Num1 leads to asymmetric mitochondria distribution shifted towards the bud [[246], [247], [248]]. Other mitochondrial tether proteins involved in inheritance are Mmr1 and Mfb1 [254,255]. Similarly, in mammalian mammary cells, PM-mitochondria contacts are important to mediate the asymmetrical distribution of mitochondria required for the epithelial-mesenchymal transition (EMT) during development [256] (Table 1). The inheritance of more mitochondria in a daughter cell is associated with increased mitochondrial fusion, a decrease in ROS and thus a more stem cell-like phenotype, driving EMT. Mechanistically, this asymmetrical distribution requires PM-mitochondria contacts, which are increased in response to EMT-inducing TGFβ1 signalling and potentially mediated by an interaction between the mitochondrial fusion protein MFN1 and the kinase PKCζ at the PM. Mitochondria are also recruited to the cleavage furrow during mammalian cytokinesis, although it is not known if interactions with the PM participate in this recruitment [257].

#### 5.2.2 Plasma membrane-mitochondria contacts function in Ca^2+^ influx

In mammalian cells, mitochondria-PM contact is suggested to function in regulating Ca^2+^ influx, specifically in relation to intracellular communications such as synaptic signalling and T cell activation [[258], [259], [260]]. In response to depletion of intracellular Ca^2+^ stores, primarily from the ER, the cell stimulates an influx of extracellular Ca^2+^ through calcium transporters at the plasma membrane, such as the calcium release-activated channel (CRAC), to restore ER Ca^2+^ stores [261]. As CRAC activity is negatively regulated by Ca^2+^, newly imported Ca^2+^ must be sequestered away from CRAC to prevent negative feedback and allow for sustained Ca^2+^ influx [262,263]. Uptake of Ca^2+^ by subplasmalemmal mitochondria has been shown to function in sequestering Ca^2+^ that enters the cell through CRAC to promote CRAC activation. Ca^2+^ influx following depletion of ER Ca^2+^ is temporally correlated with movement of mitochondria to the PM. This mitochondrial movement is dependent on extracellular Ca^2+^ and CRAC activity, indicating that mitochondria movement is a response to Ca^2+^ import specifically [264]. As well, influx of extracellular Ca^2+^ imported through CRAC is correlated with increased mitochondrial Ca^2+^ levels, indicating that influxed Ca^2+^ is shuttled into mitochondria [265]. Together these data indicate that import of Ca^2+^ into mitochondria is required for Ca^2+^ influx by preventing accumulation of Ca^2+^ accumulation near CRAC channels (Table 1; Fig. 3).

The mitochondrial calcium uniporter has a very low affinity for Ca^2+^ and can only be activated by high (μM) concentrations of Ca^2+^, far exceeding physiological concentrations [266]. It is proposed that this high concentration is achieved through proximity of the mitochondrial calcium uniporter to source calcium channels at MCSs, where microdomains of high Ca^2+^ are formed. This type of spatial coupling between calcium source channels and the mitochondria calcium uniporter at contact sites has been extensively studied at the mitochondria-ER contact site [54]. Thus, the regulation of local Ca^2+^ concentrations immediately proximal to CRAC channels by mitochondria necessitates tight proximity of mitochondria to CRAC channels, such as would be achieved by mitochondria-PM contact. In support of this, mitochondria Ca^2+^ uptake was shown to increase with increasing Ca^2+^ influx, even under conditions where global cellular Ca^2+^ content was kept constant. This suggests that mitochondria specifically respond to local Ca^2+^ levels in the proximity of CRAC channels, and that mitochondria are close enough to such channels to do so [265]. Additionally, a number of studies which spatially examined mitochondrial Ca^2+^ uptake correlated mitochondria proximity to the PM with mitochondrial Ca^2+^ import [[267], [268], [269]].

## 6 Conclusions/perspectives

After a period of describing MCSs between different organelles and the tethers involved, the field is now moving towards unraveling the diverse functions of organelle contacts and their physiological importance. As evident from the above (see also Table 1), generic physiological functions of MCSs include roles in membrane lipid exchange, channeling of metabolites, ion homeostasis and signalling, as well as organelle biogenesis and dynamics, including organelle positioning, transport and inheritance.

Although the mechanisms of lipid exchange at MCSs are not well understood, it is suggested that lipid transfer facilitates channeling of lipids to specific compartments to support metabolic processes as well as the membrane expansion of organelles such as mitochondria and peroxisomes (see 2.1.3, 2.3.2, 3.1.1, 3.4.2). Lipid transfer may also change the membrane lipid composition (e.g. to allow membrane remodeling for deformation and expansion) and modulate signalling processes at membranes (e.g. through phosphatidylinositols) (see 2.1.3, 4.1.2, 5.1.2). In addition, it has been proposed that non-vesicular lipid transfer through MCSs can compensate for an impairment of vesicular transport [270], thus linking MCSs to vesicular trafficking. In line with this, MCS also play a role in protein sorting in endosomal trafficking pathways [271].

Metabolic channeling at MCS allows efficient transfer of metabolites between compartments. MCSs may help to concentrate substrates at the organelle interface and control substrate activation (e.g. synthesis of fatty acyl-CoA) and entry into organelle-specific pathways (e.g. fatty acid β-oxidation versus fatty acid elongation at the peroxisome-ER interface) (see Section 3.1.1). This may prevent the consumption of those metabolites by other pathways, as well as their accumulation, which may have toxic effects for the cell. Metabolites that are channeled include fatty acids, ceramides and sterols but also iron and calcium (see Table 1). ER-mitochondria MCSs determine mitochondrial Ca^2+^ levels thus controlling mitochondrial functions (e.g. oxidative phosphorylation, ROS production, and apoptosis) (see Section 2.1.2).

MCSs also play important roles in cellular signalling. They facilitate the exchange of signalling molecules such as Ca^2+^, ROS and signalling lipids (e.g. phosphoinositides) (see 2.1.2, 5.1.3, 5.2.2, 2.1.4, 4.1.2, 5.1.2), often across several compartments. In this respect, the understanding of MCSs between multiple organelles is of interest (e.g. triple contacts). MCSs also serve as signalling hubs by assembling proteins involved in signal transduction, which has been linked to cellular stress responses (e.g. oxidative stress, lipid peroxidation, starvation). These conditions can trigger apoptosis and may link MCSs to age-related diseases such as neurodegeneration, cancer and Type 2 diabetes. How MCSs are altered under certain cellular stress conditions and how they may help to overcome cellular stress is of major interest in the field. Future studies will reveal what important roles MCSs may play in age-related and metabolic disorders.

Finally, MCSs regulate organelle membrane dynamics. As previously mentioned, lipid transfer allows membrane expansion and shape changes of the organelles, which are often linked to organelle biogenesis (e.g. membrane growth prior to organelle multiplication) (see 3.1.1, 3.4.2). MCS also determine sites of organelle fission, often in conjunction with the ER and actin assembly providing mechanical roles. MCSs can anchor organelles at specific cellular locations thereby regulating organelle transport, positioning and inheritance (see 3.1.2, 5.2.1). They are also involved in autophagosome formation determining organelle number and quality control.

There are still many unknowns surrounding the physiological functions, properties and regulatory mechanisms of MCSs, though our understanding is rapidly increasing. For example, an intriguing recent study has made the novel suggestion that phase properties of the ER membrane may influence the types of contact sites it makes. Using large intracellular vesicles isolated from hypotonic cell swelling and labelled to determine organelle identity, King et al. demonstrated that contacts with PM, mitochondria and endosomes occur at ER membrane subdomains characterised by ordered lipids, whereas lysosomes and peroxisomes form contacts at disordered ER membrane subdomains [272]. While the mechanism behind this remains unclear, it may represent an additional level of spatial and/or functional regulation of ER-organelle contact sites within the crowded cellular environment. Since MCSs are evidently vital for a whole host of physiological functions, the various levels and mechanisms by which they are regulated promises to be a fascinating and insightful question for the field of cell biology in the near future.

## Details of the contributions of individual authors

MS, RC and PK planned the manuscript. BS prepared the Figures. All authors contributed to the writing of the manuscript.

## Declaration of competing interest

The authors declare that they have no known competing financial interests or personal relationships that could have appeared to influence the work reported in this paper.
